# Longitudinal brain ageing after stroke: a marker for neurodegeneration and its relevance for upper limb motor outcome

**DOI:** 10.1093/braincomms/fcaf299

**Published:** 2025-08-14

**Authors:** Raphael B Takyi, Jeanette Plantin, Sylvain Charron, Marc A Maier, Jean-Claude Baron, Guillaume Turc, Charlotte Rosso, Clément Debacker, Påvel G Lindberg

**Affiliations:** Université Paris Cité, Institute of Psychiatry and Neuroscience of Paris, INSERM U1266, F-75014 Paris, France; Department of Clinical Science, Karolinska Institutet, Danderyd University Hospital, 171 77 Stockholm, Sweden; Université Paris Cité, Institute of Psychiatry and Neuroscience of Paris, INSERM U1266, F-75014 Paris, France; Department of Radiology, GHU-Paris Psychiatrie et Neurosciences, F-75014 Paris, France; Université Paris Cité, CNRS, Saints-Pères Paris Institute for the Neurosciences, F-75006 Paris, France; Université Paris Cité, Institute of Psychiatry and Neuroscience of Paris, INSERM U1266, F-75014 Paris, France; Department of Neurology, GHU-Paris Psychiatrie et Neurosciences, F-75014 Paris, France; Université Paris Cité, Institute of Psychiatry and Neuroscience of Paris, INSERM U1266, F-75014 Paris, France; Department of Neurology, GHU-Paris Psychiatrie et Neurosciences, F-75014 Paris, France; Sorbonne Université, INSERM U1127, CNRS UMR 7225, STARE Team, iCRIN, Institut du Cerveau, APHP Urgences CérebroVasculaires, Hôpital Pitié-Salpêtrière, 75013 Paris, France; Université Paris Cité, Institute of Psychiatry and Neuroscience of Paris, INSERM U1266, F-75014 Paris, France; Department of Radiology, GHU-Paris Psychiatrie et Neurosciences, F-75014 Paris, France; Université Paris Cité, Institute of Psychiatry and Neuroscience of Paris, INSERM U1266, F-75014 Paris, France; Department of Clinical Science, Karolinska Institutet, Danderyd University Hospital, 171 77 Stockholm, Sweden

**Keywords:** stroke, brain age, voxel-based morphometry, upper limb, motor outcome

## Abstract

Brain age, as distinct from chronological age, may reveal post-stroke recovery mechanisms, but longitudinal studies tracking brain age are lacking. We explored longitudinal change of brain age post-stroke and its relation to upper limb sensorimotor outcome. T_1_-weighted MRI at baseline (∼3 weeks) and follow-up (3–7 months) post-stroke was used to estimate brain age. Difference to chronological age was calculated as brain age gap (BAG). Grey and white matter changes and lesion location related to increased brain ageing were investigated, controlling for lesion volume. Association between BAG change and upper limb sensorimotor outcome was studied using linear mixed effects regression. Totally, 114 stroke patients with arm/hand hemiparesis were pooled from three studies. BAG significantly increased from baseline to follow-up, a period of ∼6 months, by a mean of 3.62 years (*t* = −7.31; *P* < 0.001). Voxel-based morphometry showed that high BAG change was related to reduced grey and white matter volume ipsilesionally, extending beyond the stroke lesion. Voxel-based lesion symptom mapping showed that lesion to thalamocortical projections, internal capsule and corona radiata related to accelerated brain ageing. BAG change was significantly associated with motor outcomes in the sub-acute to chronic phase, as expressed by Fugl–Meyer assessment (*β* = −5.62, SE = 2.81, *t* = −2.00, *P* = 0.05), maximum grip strength (*β* = −0.14, SE = 0.04, *t* = −3.36, *P* = 0.001) and dexterity assessment (*β* = −0.09, SE = 0.04, *t* = −2.17, *P* = 0.03). We demonstrate increased brain ageing within the first few months post-stroke. This secondary neurodegeneration was negatively related to motor outcome. Brain age may be a valid whole-brain probe of individual secondary post-stroke degeneration, relevant for predicting recovery and identifying targets of neural plasticity.

## Teaser

Takyi *et al*. report brain age changes in months following stroke reflecting neurodegeneration, particularly in ipsilesional thalamus. Brain age change was a significant predictor of post-stroke hand motor function. These findings support brain age as a marker of neurodegeneration with potential implications for recovery prediction and patient stratification in clinical trials.

## Graphical Abstract

**Graphical Abstract:**
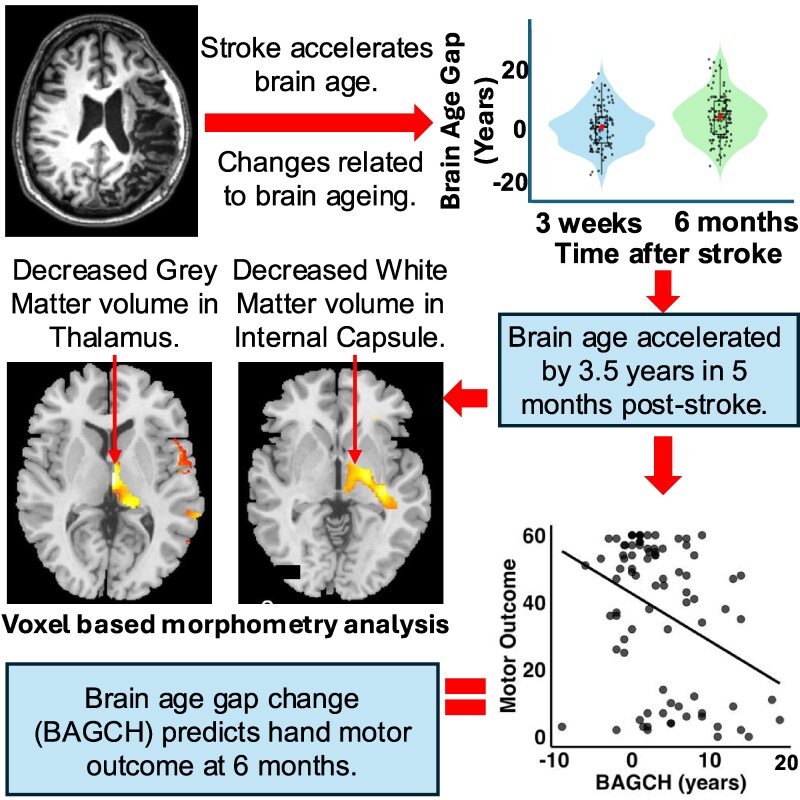

## Introduction

Brain age assesses the overall health and integrity of the brain. It is computed by analysing anatomical MRI images with machine learning or deep learning algorithms, and the difference to chronological age is computed as brain age gap (BAG). A negative BAG indicates a younger brain, while a positive BAG indicates an older brain relative to the chronological age.^1,2^ An important step in brain age calculation is a normalization procedure,^3^ which can be inadequate in the presence of brain lesions such as in stroke. Previous studies^4,5^ have circumvented this problem by using enantiomorphic normalization,^6^ which replaces the lesioned tissue with the corresponding healthy contralesional tissue. Subsequently, cross-sectional studies in stroke have associated older brain age to stroke,^4,7^ but this effect could be due to early life factors rather than longitudinal brain age changes,^8^ necessitating the need for longitudinal studies to observe brain ageing trajectories post-stroke. A recent longitudinal study in small-volume stroke lesions found a 1-year increase in brain age between baseline and 6-month follow-up.^9^ However, this study did not address brain age changes in stroke patients with larger lesions, which has been undertaken in the current longitudinal study.

Age-related grey (GM) and white matter (WM) volume decline is well documented.^10^ Concurrently, studies have demonstrated that stroke leads to a reduction in GM and WM volume.^11,12^ This underscores the notion that accelerated brain ageing in stroke is partly attributable to secondary loss of GM and WM volume. Voxel-based morphometry (VBM) has been used to identify primary lesion sites that are related to secondary GM volume reduction post-stroke, with the thalamus being a vulnerable area.^11,13,14^ This is likely due to the widespread reciprocal thalamocortical connections,^15,16^ which are involved in complex processing, and whose lesion exerts extensive secondary neurodegeneration. A recent study reported a large variability of brain age in stroke patients, i.e. from 24 below to 22 years above chronological age.^17^ Given this variability in brain age among stroke patients, the extent of degeneration between individuals with high and low brain age is likely to differ, a phenomenon that is not well studied. VBM is a method of comparing the GM volume of two groups,^18^ and thus can be used to study GM degeneration in stroke patients who fall on either side of the brain age spectrum. Another concept within the domain of brain age in stroke that is less understood is the identification of lesion locations that drive accelerated brain age. Voxel-based lesion symptom mapping (VLSM) is a method that assesses the voxel-wise association between lesion location and behavioural measures.^19,20^ This technique can also be utilized to identify lesion locations that are associated with other (non-behavioural) measures and variables, such as accelerated brain age in stroke patients.

Some longitudinal studies in stroke patients have shown a correlation between increased brain age and cognitive outcomes.^17,21^ Even though motor deficits remain the most prevalent post-stroke impairment,^22^ studies that have investigated the association between brain age and sensorimotor outcomes^2,23^ have been predominantly cross-sectional. These need to be substantiated by longitudinal investigations of brain age in relation to motor outcome. Specifically, no study has assessed the relationship between brain age and post-stroke hand motor function, including grip strength and manual dexterity, which are essential for daily activities.^24,25^

In this study, we sought to describe the longitudinal changes in brain age observed in patients with moderate to severe stroke and larger lesions during the initial 3-to-7-month period post-stroke. We hypothesized that brain age of stroke patients would markedly increase in the months following stroke, reflecting secondary neurodegeneration extending beyond the primary stroke lesion. We then investigated underlying mechanisms of brain age alterations. First, we studied whether structural brain changes relate to brain age changes through a VBM analysis. We predicted that patients with high brain age would show stronger GM and WM atrophy distant to the lesion compared to patients with younger brain age. Second, VLSM was used to study whether lesion location could predict longitudinal brain age changes. Third, we also investigated brain age in relation to post-stroke sensorimotor outcome. We hypothesized that higher change (increase) of brain age post-stroke would be associated with poor upper limb motor outcomes, and particularly so for manual dexterity, which is strongly affected by chronological ageing.^26^

## Materials and methods

### Study design and participants

Longitudinal data, i.e. T_1_-weighted MRI scans and clinical scores at the initial (∼3 weeks post-stroke) and the follow-up time point (3–7 months), were pooled from three studies^27-29^ (for comprehensive account of methodologies, see these three studies). Inclusion criteria were: (i) first ever ischaemic or haemorrhagic stroke in supratentorial brain areas, (ii) persistent upper limb weakness measured by either Fugl–Meyer Assessment or the National Institute of Health Stroke Scale (NIHSS), and (iii) older than 18 years. Patients with the following characteristics were excluded: (i) contraindication to MRI, (ii) severe aphasia or inability to follow a rehabilitation program and (iii) suffering from other life-threatening conditions. Each study received ethical approval from the local ethics board (see Study C: Jacquemont *et al*., Study B: Rosso *et al*., and Study A: Plantin *et al*.^27-29^ ) and all patients provided written informed consent. T_1_-weighted MRI data and motor scores were obtained at about 3 weeks (baseline) of the initial stroke episode, and at follow-up at 3 months,^27^ or 6 months^28,29^ (follow-up) (Table 1).

**Table 1:** Characteristics of the three study cohorts (A, B and C)

	ALL	Study A	Study B	Study C
Total	114	71	28	15
Age (years)	55.08 ± 11.96	52.82 ± 8.85	56.31 ± 16.14	63.53 ± 12.47
Female, *N* (%)	35 (30.7%)	19 (26.8%)	10 (35.7%)	6 (40%)
Stroke type (*N* ischaemic, %)	90 (78.95%)	51 (72.86%)	28 (100%)	11 (73.33%)
Lesion volume (cm^3^)	78.32 ± 126.63	113.65 ± 149.80	22.10 ± 31.64	16.02 ± 23.13
NIHSS		8 ± 5	4 ± 4	NA
FMA-UE (max = 66)	24.78 ± 21.74	23.96 ± 22.67	NA	28.67 ± 16.75
Dext [0, 1], 1 = max	0.21 ± 0.28	0.21 ± 0.28	NA	NA
mGS	0.37 ± 0.41	0.30 ± 0.38	0.60 ± 0.46	0.28 ± 0.34
Days post-stroke (days)	25.81 ± 9.09	25.20 ± 6.67	27.54 ± 9.02	25.47 ± 16.72
Days between scans (days)	158.61 ± 44.96	156.14 ± 15.40	203.71 ± 52.77	86.13 ± 5.83
Time to follow-up scan (T2, months)	5.65 ± 1.59	6.12 ± 0.30	7.32 ± 0.32	3.52 ± 0.31

NIHSS, National Institute of Health Stroke Scale (scores of 5–14 correspond to mild to moderately severe); FMA-UE, Fugl–Meyer assessment of the upper extremity; mGS, maximum grip strength (ratio of less affected to affected side); Dext, strength-dexterity score; NA, not available.

Data were obtained at baseline (TP1), except for the clinical motor scores obtained at follow-up (TP2, 3–7 months). All values are shown as mean ± SD.

### MRI acquisition

Brain imaging for both time points of study A^29^ was performed with an Ingenia 3.0T MRI scanner (Philips, Cambridge, MA, USA) with an eight-channel head coil. High-resolution T_1_-weighted anatomic images were acquired with turbo field echo 3D sequence: field of view 250 × 250 × 181 mm, matrix 228 × 227, slice thickness 1.2 mm, slice spacing 0.6 mm and number of slices 301 (echo time, shortest; relaxation time, shortest). Images from both time points of the two other studies (B & C)^27,28^ were acquired with a 3T Siemens Trio MRI scanner. A 12-channel head matrix coil was used. The MRI protocol included 3D-T1 SPGR (TR = 2.3 s; TE = 4.18 ms; flip angle = 9°; TI = 900 ms; matrix = 240 × 256; voxel size = 1 × 1 × 1 mm^3^; 176 slices).

### Lesion analysis

Lesion masks were manually delineated by two raters (R.B.T. and J.P.) on all native space T_1_-weighted axial slices using MRIcron (http://people.cas.sc.edu/rorden/mricron/index.html) and verified by an experienced neurologist (J-C.B.). Lesion volumes showed excellent inter-rater reliability (interclass correlation coefficients = 0.91). All left-sided lesions were flipped to the right for group analysis. The lesion masks were used in the process of enantiomorphic normalization^6,30^ to replace the lesioned tissue prior to brain age calculation. Briefly, a mirrored version (enantiomorphic image) of the non-lesioned hemisphere is generated and serves as a surrogate for the lesioned hemisphere. The lesioned area in the original image is replaced with the corresponding region from the mirrored image, thereby creating a pseudo-complete brain image. The modified (enantiomorphic) image is used for spatial normalization, ensuring accurate alignment with standard templates without distortion caused by the lesion. This approach enables reliable normalization while preserving anatomical accuracy and avoiding biases from lesion-related deformations. We used the MR segment-normalize function implemented in the SPM12 clinical toolbox and specified two inputs for the normalization procedure: the T_1_-weighted MRI and its corresponding lesion mask.^30^ Lesion maps were analysed to compute the weighted corticospinal tract lesion load (wCST-LL, measured in cm^3^) using a predefined CST template.^31^ This template incorporated regions of interest in the precentral gyri, posterior limb of the internal capsule, cerebral peduncle and anteromedial pons. WM hyperintensities (WMHs) were segmented using the SAMSEG tool, integrated within FreeSurfer (version 7.2).^32,33^ SAMSEG is an atlas-based probabilistic segmentation algorithm that enables the automated identification of brain structures. The SAMSEG pipeline was applied to the T_1_-weighted images to perform tissue segmentation. WMHs were segmented as part of the WM tissue class based on intensity variations within the T_1_-weighted images, guided by the probabilistic atlas provided with SAMSEG. Segmentation volumes for WMHs were quantified in cubic millimetres.

### Brain age calculation

Brain age was calculated with the brainageR pipeline (https://github.com/james-cole/brainageR). The software package utilizes a Gaussian Processes regression implemented in R (https://www.r-project.org) to generate a predicted age value from a T_1_-weighted MRI scan using SPM12 for segmentation and normalization (Crinion *et al*., 2007). Briefly, GM, WM and cerebrospinal fluid (CSF) probabilistic tissue maps are concatenated, vectorized and undergo dimensionality reduction using principal component analysis (PCA). PCA is run using R’s prcomp, and the top 80% of the variance are retained. The rotation matrix of the PCA is then applied to new MRI data, and the resulting PCA matrix is used to predict brain age value using the regression analysis implemented in the kernlab package in R. Version 2.1 of the brainageR software was used for our brain age calculation. For that version, training was carried out on 3377 healthy individuals from seven publicly available datasets and tested on two independent datasets.^1^ The mean age of the individuals was 40.6 ± 21.4 years, with a range of 18–92 years. We then calculated BAG as


BAG=EstimatedBrainAge−ChronologicalAge

BAG < 0 indicates a relatively younger brain age relative to the chronological age and vice versa. After that we calculated BAG change (BAGCH) as


BAGCH=BAG(follow-up)−BAG(baseline)

### Voxel-based lesion symptom mapping

Individual normalized T_1_-weighted stroke lesions were used for VLSM to investigate the relationship between lesion location and BAGCH using the NiiStat toolbox (https://www.nitrc.org/projects/niistat/).^20^ Voxels were identified as belonging to lesioned tissue if at least 10 patients were included. The location of WM voxels correlating with BAGCH were analysed using the John Hopkins University white matter atlas.^34^

### Voxel-based morphometry

VBM analysis was performed on enantiomorphic normalized T_1_-weighted MRIs using Computational Anatomy Toolbox (CAT12) (http://dbm.neuro.uni-jena.de/cat),^35^ an extension toolbox of Statistical Parametric Mapping (SPM12, http://www.fil.ion.ucl.ac.uk/spm/software/spm12). To account for within-subject variability, the CAT12 longitudinal pipeline creates an intra-subject template by averaging scans across time points. This approach reduces noise from scanner differences and alignment errors, enabling precise detection of subtle changes in GM, WM and CSF volumes. Default settings of the longitudinal module, optimized for detecting large changes such as those associated with ageing were used. Location of GM areas outside of the lesion mask with notable volume loss between the two time points were identified using the automated anatomical labelling atlas.^36^ WM regions were identified using the John Hopkin University WM atlas.^34^

### Clinical outcome measures

The clinical motor measures of the patients included Fugl–Meyer assessment of upper extremity (FMA_UE), which was assessed in two of the studies (Studies A, C); strength-dexterity test (DextSc), which was assessed in Study A only; and the maximum hand grip strength (expressed as ratio of less affected to affected side, mGS), which was assessed in all three studies. mGS reflects the degree of post-stroke weakness and is highly related to the degree of corticospinal tract damage.^28^ FMA_UE reflects upper limb motor impairment.^37^ Contralesional DextSc, consisting of compressing a spring between index and thumb, assesses dynamic precision grip control and was included because of evidence for reduced manual dexterity even in patients with little to no clinical upper limb motor impairment.^38^ These measures have been previously detailed.^24,28,29,38^ These measures were not available for all patients (Table 1), so analyses were based on the number of patients assessed with each measure.

### Statistical analysis

Normality and skewness of data were checked prior to analysis using Shapiro–Wilk test (and skewness >1 considered as substantially skewed). We used Pearson's correlation coefficients to compare relations between brain age and chronological age over the two time points. In addition, descriptive statistics included mean values, standard deviation, mean absolute error, and *R*^2^.

To evaluate whether brain age increased significantly from the sub-acute to the chronic phase, we used a paired *t*-test to compare BAG at baseline and follow-up. We also performed correlation analysis to ascertain whether lesion volume influences BAG.

For VBM analyses, all T_1_-weighted MRIs with lesions in the left hemisphere were flipped to the right, in accordance with the standard methodology employed in such analyses. We first examined the relationship between GM and WM volumes at baseline, follow-up, and over time controlling for baseline BAG and lesion volume. Next, to identify with enhanced sensitivity the brain areas that showed secondary atrophy post-stroke, we calculated the median of BAGCH and grouped patients into two groups. Those above the median were classified as having older brains (high BAGCH), whereas those below the median were classified as having younger brains (low BAGCH). Two-sample *t*-tests were performed to assess group differences between the low and high BAGCH groups in terms of chronological age, lesion volume and baseline motor scores. Longitudinal flexible factorial design in CAT12 was used to examine the difference in GM and WM volumes in the two groups across the two time points using statistical threshold of family-wise error (FWE) = 0.05 and cluster threshold of 100 voxels.

We performed VLSM analysis to identify associations between lesion location and BAGCH. Lesions maps were normalized using Montreal Neurological Institute (MNI) template, and statistical comparisons were performed on a voxel-wise basis. Continuous BAGCH values were used as dependent variables and analysis restricted to voxels lesioned in at least 10 patients. To control for potential confounding effect of lesion size, lesion volume was included as a covariate in the analysis. We applied the permutation method (3000 repetitions) using a whole-brain general linear model to identify voxels where the lesion was related to BAGCH (corrected for false discovery rate: *P*_FDR_ < 0.05).

We performed a linear mixed-effect regression analysis with BAGCH as the primary predictor and study cohort as a random effect. This approach was employed to account for any differences that may have existed among the three study cohorts. Chronological age was used as a covariate due to a well-documented regression dilution effect.^39,40^ Other covariates used included the baseline BAG scores, days post-stroke, days between scans, sex, total intracranial volume (TIV) and WMHs. Because left-sided lesions were flipped to the right for VBM analysis, we included lesion side also as a covariate. Predictors were standardized (*z*-scored) to facilitate comparison of effect sizes and address scaling differences. Outcome variables included FMA_UE, mGS and DextSc. Statistical significance was set at *P* ≤ 0.05. All analyses were conducted using R programming language version 2023.06.0+421.

## Results

**Figure 1:**
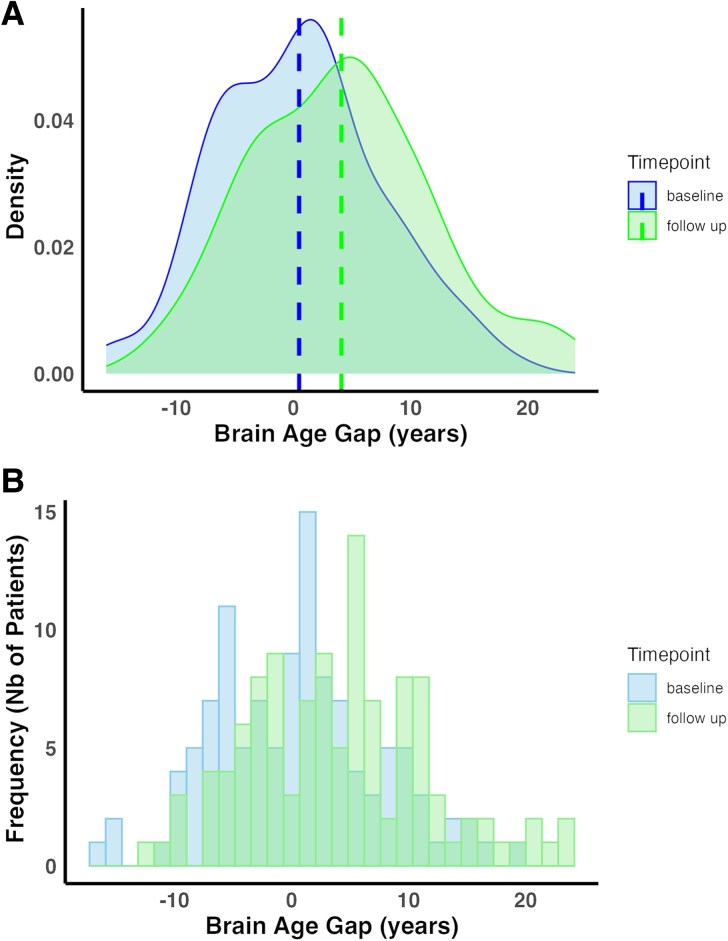
**Distribution of BAG at baseline and follow-up.** (**A**) Density plot showing the overlapping distribution of BAG at baseline and follow-up. The peak of BAG at follow-up is older than the peak at baseline. Vertical stippled lines indicate the mean of BAG at baseline and follow-up respectively (*N* = 114). (**B**) Frequency plot showing the overlapping distribution of BAG at baseline and follow-up. The peak of BAG at follow-up is older than the peak at baseline (*N* = 114). Nb, Number.

MRI and outcome data were analysed in 114 participants (31% females) with an average age of 55.08 ± 11.96 years (clinical characteristics listed in Table 1). Most of the participants (79%) had ischaemic lesions. A majority of the lesions (52.6%) were right-sided. The average lesion volume was 78.32 ± 126.63 mL at baseline. The average brain age at baseline was 55.56 ± 12.84 years with a mean absolute error of 5.61 years (Table 2). BAG was not significantly correlated to chronological age (*r* = −0.16, *P* = 0.08).The median BAGCH between baseline and follow-up, a period of 5.7 months on average (Table 2), was 2.3 years. There were significant correlations between brain age and chronological age at baseline and follow-up (Table 2, Supplementary Figs. 1 and 2). The distributions of BAG at baseline and follow-up are shown in Fig. 1. Supplementary Figs 3 and 4 show the distribution of BAGCH and the longitudinal BAGCH for each participant, respectively. At baseline, 48 participants had negative BAG as compared to 66 who had positive BAG. At follow-up, 36 participants had negative BAG and 78 had positive BAG. Over the two time points, 84 participants showed an increase in BAG, whereas 22 experienced a reduction in BAG. Only eight participants experienced no change in BAG over time.

**Table 2:** Correlations between chronological age and estimated brain age at TP1 and TP2 (*N* = 114)

Time point	Brain age (years) (mean ± SD)	*r*	*P*-value	*R* ^2^	Mean absolute error	BAG (years) (mean ± SD)
TP1	55.56 ± 12.84	0.84	<0.001	0.71	5.61	0.48 ± 7.02
TP2	59.17 ± 12.40	0.80	<0.001	0.63	6.98	4.09 ± 7.78

TP1, baseline (∼3 weeks post-stroke); TP2, follow-up (3–7 months); *r*, Pearson’s correlation coefficient; *R*^2^, coefficient of determination (% variance explained); mean absolute error, absolute difference in years when subtracting chronological age from estimated brain age (i.e. ≠ BAG).

**Figure 2:**
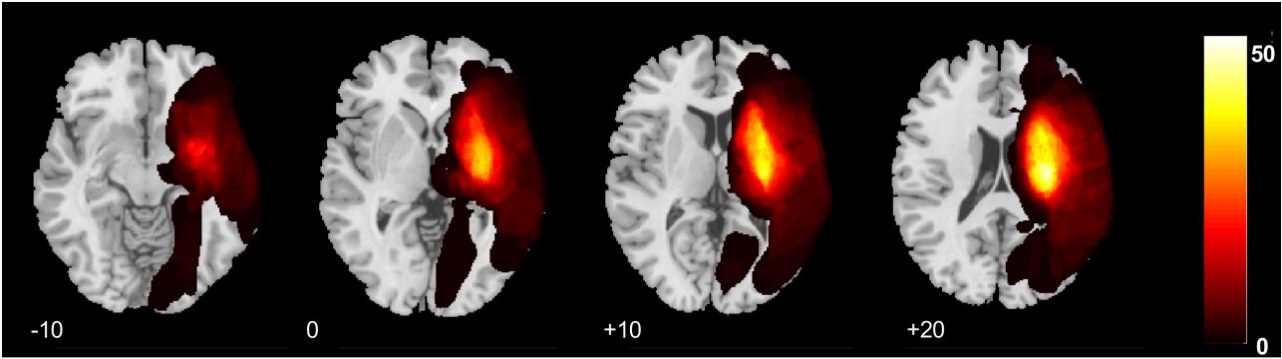
**Lesion overlap map of the study cohort.** Overlapping lesion map of the study cohort on an MNI template. Numbers correspond to the coordinate of the axial slice in the MNI space. Colour scale bar: number of patients with lesions in a particular region. The lesion outline of each patient was based on T_1_-weighted MRI acquired in the sub-acute stage. The right side of the images represents the right side of the brain (here and in the following MRI Figures) (*N* = 114).

The localization of the stroke lesion among the patients is shown in Fig. 2. There were no statistically significant differences observed between the high and low BAGCH groups in chronological age (*t* = −0.99, *P* = 0.39) and lesion volume (*t* = −0.28, *P* = 0.80). Neither was there in baseline motor score: FMA (*t* = −0.12, *P* = 0.92), mGS (*t* = −0.23, *P* = 0.83) or dexterity (*t* = −0.94, *P* = 0.44). BAGCH was significantly correlated to wCST-LL (*r* = 0.39, *P* < 0.001).

### Brain age is increased from the sub-acute to the chronic phase

The mean BAG at baseline was 0.53 ± 7.03 years but increased to 4.15 ± 7.78 years at follow-up, i.e. ∼6 months later. Paired *t*-test revealed a significant difference between BAG at baseline and follow-up (mean difference = 3.62 years; *t* = −7.31; *P* < 0.001). There were also significant positive correlations between BAG and lesion volume at both baseline (*r* = 0.19, *P* = 0.05) and follow-up (*r* = 0.35, *P* < 0.001).

### Change of BAG over time and brain atrophy

**Figure 3:**
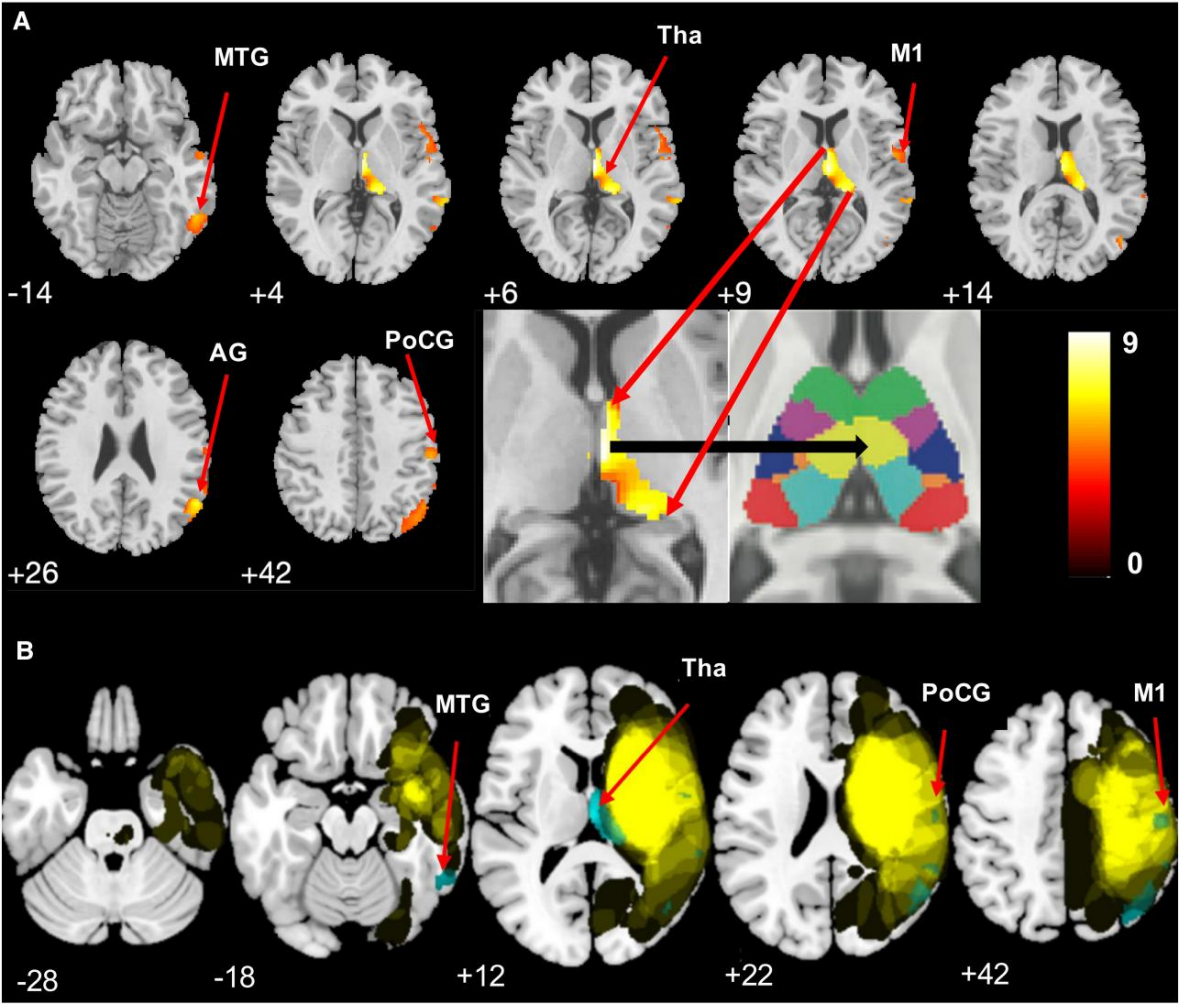
**VBM analysis showing GM loss in patients with high change in BAG.** (**A**) VBM analysis showing regions of decreased GM volume in patients with high change (increase) in BAG. Colour scale bar: magnitude of *t-*values. Inset: maximal likelihood template *Z* = 7 (adapted from Najdenovska et al., 2018, licensed under CC BY 4.0.) showing the location of the peak thalamus effect. MTG, middle temporal gyrus; Tha, thalamus; M1, primary motor area; AG, angular gyrus; PoCG, postcentral gyrus. (**B**) Overlap (yellow) of the lesion maps and clusters of significant GM loss (cyan) outside the lesion on an MNI template. Numbers correspond to the Z-coordinate of the axial slice in the MNI space. All t-values at *P*_FWE_ < 0.05 (*N* = 57).

**Figure 4:**
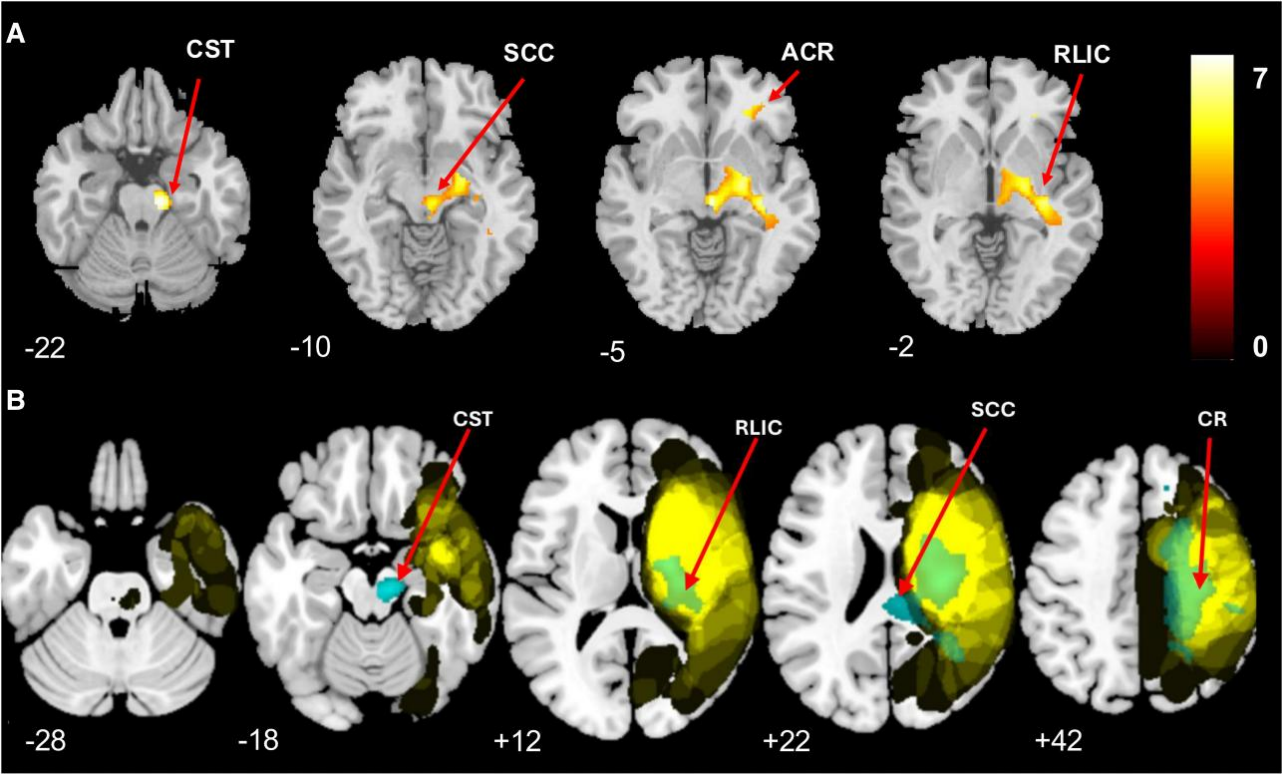
**VBM analysis showing WM loss in patients with high change in BAG.** (**A**) VBM analysis shows regions of decreased WM volume in patients with high change (increase) in BAG. Mainly affected are internal capsule (anterior and posterior limb) and superior and posterior corona radiata. Colour scale bar: magnitude of *t-*values. CST, corticospinal tract; SCC, splenium of corpus callosum; ACR, anterior corona radiata; RLIC, retro-lenticular part of internal capsule; CR, corona radiata. (**B**) Overlap (yellow) of the lesion maps and clusters of significant WM loss (cyan) outside the lesion on an MNI template. Numbers correspond to the Z-coordinate of the axial slice in the MNI space. All *t*-values at *P*_FWE_ < 0.05 (*N* = 57).

We first examined the relationship between GM and WM volume and BAG at baseline and follow-up. There was a significant relationship between BAG and GM volume, with high BAG related to reduced volume in right thalamus (*t* = 4.10, *P*_UNCORR_ < 0.001), in right middle temporal gyrus (*t* = 4.18, *P*_UNCORR_ < 0.001) at baseline and in right thalamus (*t* = 3.78, *P_UNCORR_* < 0.001) at follow-up. Similarly, there was significant WM volume reduction related to high BAG in left cerebellar WM (*t* = 3.42, *P*_UNCORR_ < 0.001) at baseline and left (*t* = 4.12, *P_UNCORR_* < 0.001) and right (*t* = 4.15, *P*_UNCORR_ < 0.001) cerebellar WM at follow-up (Supplementary Fig. 5). We then studied the association between GM and WM volumes over the two time points controlling for baseline BAG and lesion volume. There was a significant GM volume reduction in right thalamus (*t* = 9.86, *P*_FWE_ < 0.05) and right insula (*t* = 8.40, *P*_FWE_ < 0.05). Other significant clusters are shown in Supplementary Table 2 and Supplementary Fig. 6. There was significant reduction in both left and right hemisphere WM volume in several regions over time (all *P*_FWE_ < 0.05) (Supplementary Table 3 and Supplementary Fig. 7). We then investigated the longitudinal effect of time on GM and WM volumes between the two groups (low versus high BAGCH). VBM analysis showed ipsilesional areas with marked reduction in GM volume from the sub-acute to chronic phase of stroke in the subset of patients with high BAGCH but not for patients with low BAGCH: GM atrophy was seen in both motor and sensory brain areas, particularly in the thalamus with a notable decrease of GM volume in the medio-dorsal nucleus (*t* = 9.22, *P*_FWE_ = 0.05) (Table 3 and Fig. 3). The MNI coordinates of all regions with significant volume reduction (all FWE = 0.05) are shown in Table 3. Several WM areas showed atrophy (Supplementary Table 1 and Fig. 4), including the corpus callosum (*t* = 6.29, *P*_FWE_ < 0.05).

**Table 3:** VBM analysis of areas of GM volume reduction in the patient group with high BAGCH

Location (Broadman area)	No. of voxels	*t*-value	MNI coordinate (mm)		
			*x*	*y*	*z*
Thalamus (medio-dorsal)	1427	9.22	2	−15	6
Angular gyrus (BA39)	1423	7.23	54	−57	26
Middle temporal gyrus (BA21)	1976	6.38	66	−40	4
Middle temporal gyrus (BA22)	106	5.05	58	−8	−14
Superior frontal gyrus (BA6)	280	5.89	27	−6	62
Rolandic operculum (BA40)	120	5.71	57	−18	14
Postcentral gyrus (BA1)	257	5.70	56	−21	42
Primary motor cortex (BA4)	429	5.68	52	−6	9

### Lesion location affects longitudinal brain ageing

**Figure 5:**
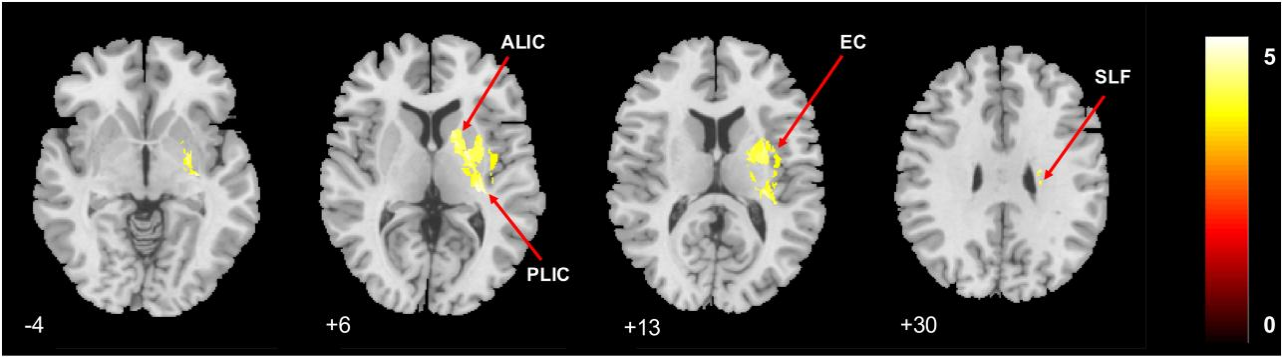
**VLSM applied to brain age.** VLSM applied to brain age shows lesioned voxels relating to high BAG on an MNI template. Numbers correspond to the Z-coordinate of the axial slice in the MNI space. These voxels were located within ALIC (anterior limb of internal capsule), PLIC (posterior limb of internal capsule), EC (external capsule), SLF (superior longitudinal fasciculus) and in other areas (Supplementary Table 4). Colour scale bar represents *t*-values. All *t*-values at *P*_FDR_ < 0.05 (*N* = 114).

We used VLSM to study the relationship between lesion site and WM areas of marked neurodegeneration. VLSM revealed that lesions to WM areas, primarily to the internal capsule (anterior limb: *t* = 5.42, *P*_FDR_ < 0.05; posterior limb: *t* = 5.42, *P*_FDR_ < 0.05) as well as to other structures (Fig. 5 and Supplementary Table 4), showed associations with high BAGCH.

### High change in BAG post-stroke is associated with worse sensorimotor outcomes

Out of the 86 patients who had FMA_UE assessments, linear mixed-effect regression analysis showed that BAGCH had a statistically significant negative association with FMA_UE scores at follow-up: FMA (*β* = −5.62, SE = 2.81, *t* = −2.00, *P* = 0.05), i.e. increased BAGCH led to lower (worse) outcome. None of the other predictors showed significant associations with FMA_UE at follow-up (all *P*-values >0.05) (Table 4).

**Table 4:** Prediction of chronic post-stoke sensorimotor outcome

Outcome variable	Predictor	*β*	SE	*t-*value	*P-*value
FMA_UE *N* = 86 *R*^2^ = 0.19	**BAGCH**	**−5**.**62**	**2**.**81**	**−2**.**00**	**0**.**05**
	Age	−2.69	3.27	−0.82	0.41
	Sex	1.52	2.84	0.54	0.59
	Days post-stroke	−3.96	2.56	−1.55	0.13
	Days between scans	−4.38	3.62	−1.21	0.23
	TIV	−1.38	3.36	−0.41	0.68
	WMH	−0.37	2.77	−0.13	0.89
	Lesion side	2.04	2.58	0.79	0.43
	Baseline BAG	0.28	2.92	0.10	0.92
mGS *N* = 114 *R*^2^ = 0.19	**BAGCH**	**−0**.**14**	**0**.**04**	**−3**.**36**	**0**.**001**
	Age	0.003	0.04	−0.08	0.94
	Sex	0.03	0.05	0.70	0.49
	Days post-stroke	−0.03	0.04	−0.73	0.47
	**Days between scans**	**0**.**09**	**0**.**04**	**2**.**40**	**0**.**02**
	TIV	0.01	0.05	0.17	0.87
	WMH	0.02	0.04	0.52	0.61
	Lesion side	0.03	0.04	0.70	0.49
	Baseline BAG	−0.07	0.04	−1.70	0.09
DextSc *N* = 71 *R*^2^ = 0.19	**BAGCH**	**−0**.**09**	**0**.**04**	**−2**.**17**	**0**.**03**
	Age	−0.02	0.05	−0.30	0.77
	Sex	0.03	0.05	0.55	0.58
	Days post-stroke	−0.05	0.06	−0.86	0.39
	Days between scans	0.04	0.11	0.35	0.73
	TIV	−0.002	0.05	0.04	0.97
	WMH	−0.002	0.04	−0.06	0.95
	Lesion side	0.03	0.04	0.85	0.40
	Baseline BAG	−0.05	0.05	−1.01	0.32

Significant predictors are shown in bold.

BAGCH, BAG change; FMA-UE, Fugl–Meyer assessment of upper extremity; mGS, maximum grip strength; DextSc, strength-dexterity test (contralesional); TIV, total intracranial volume; WMHs, WM hyperintensities; Beta, standardized regression coefficient for each predictor variable; SE, standardized error.

Similarly, linear mixed-effect regression analysis showed that BAGCH had a statistically significant negative association with mGS scores at follow-up (*β* = −0.14, SE = 0.04, *t* = −3.36, *P* = 0.001). The number of days between scans was also significantly associated with mGS at follow-up (*β* = 0.09, SE = 0.04, *t* = 2.40, *P* = 0.02). None of the other predictors significantly correlated with mGS at follow-up (all *P*-values >0.05) (Table 4).

For the subset of the cohort that had manual dexterity measures, BAGCH had a statistically significant negative association with dexterity (DextSc) scores at follow-up (*β* = −0.09, SE = 0.04, *t* = −2.17, *P* = 0.03). Again, none of the other predictors significantly correlated with DextSc at follow-up (all *P*-values >0.05) (Table 4).

In a separate analysis of motor outcome prediction, where we included baseline motor scores, baseline motor score explained over 70% of the variance of motor outcome at follow-up. Under these conditions, BAGCH was not significant in predicting motor outcome at follow-up (Supplementary Table 5).

## Discussion

In this longitudinal study, we demonstrate that brain age increases following stroke, and there is a significant difference in GM and WM volume loss between patients with a high change (increase) in BAG and patients with a low change in BAG. We also show that brain age change, rather than chronological age, is a predictor for motor outcome.

To better understand how stroke accelerates brain age, longitudinal brain age studies are needed.^8^ A recent longitudinal study in a population of stroke patients with smaller lesions found an increase in brain age of approximately 1 year over a 6-month period.^9^ Compared to this previous study, in which patients had an average lesion volume of 5 cm^3^, our study reveals that this increase in brain age is even bigger in patients with moderate to severe stroke, who typically have larger lesions (average lesion volume was 78 cm^3^): brain age increased by about a 10-fold factor. Our analysis showed that between baseline and follow-up (3–7 months post-stroke), BAG increased by approximately 4 years, i.e. overproportionately. We also found significant positive correlations between BAG and lesion volume, implying that the larger the lesion, the higher the BAG. The association between BAG and lesion volume has not been reported consistently: some studies reported no relations,^4^ whereas others found significant associations.^23^ However, there may be a threshold effect such that mild strokes with small lesions tend to not influence motor function,^41^ whereas larger lesions more likely affect a significant portion of GM and WM regions,^42^ which in turn alters brain age estimation.^43,44^ Therefore, lesion volume is more likely than not to increase BAG and impair motor function.

Previous cross-sectional studies have shown that stroke induces secondary neurodegeneration months after the initial stroke episode, and this happens in various brain areas.^12,45^ We first used VBM to study the cross-sectional reduction of GM and WM volume at baseline and follow-up. There was a significant GM volume reduction related to high BAG in the right middle temporal gyrus at baseline and in the right thalamus at baseline and follow-up, respectively. WM volume reduction, related to high BAG, was found in the left cerebellar WM at baseline and at left and right cerebellar WM at follow-up. We then examined longitudinal GM and WM volume reduction in the whole group and found significant volume reduction in diverse motor and sensory brain areas. In the subsequent analysis, after applying a median split to the group, we found differential secondary neurodegeneration between patients showing high versus low BAGCH: patients with high change (increase) in BAG experienced GM and WM volume reduction over time, contrasting with stable GM and WM volumes in patients with low change in BAG. Importantly, brain age did not accelerate homogenously throughout the brain after stroke: based on a maximum probability atlas,^46^ brain regions with marked degeneration included the thalamus, cortical structures and WM tracts. First, the medio-dorsal nucleus of the thalamus, a high-order relay thalamic nucleus,^15^ with strong connections to the prefrontal cortex,^16^ showed degeneration. This nucleus, implicated in memory and cognition,^16,47^ has also been associated with motor learning,^48^ and likely contributes to motor recovery after stroke.^49^ Second, patients with high BAGCH showed degeneration in other cortical areas, including the middle temporal gyrus, superior frontal gyrus, angular gyrus, rolandic operculum, postcentral gyrus and primary motor cortex. These interconnected areas show age-dependent degeneration in healthy subjects^50,51^ and are likely to be affected by stroke-induced disconnections. Third, a marked volume reduction was observed among multiple WM tracts in stroke patients with high BAGCH, including the corona radiata, internal capsule, superior longitudinal fasciculus, cingulum and corpus callosum. These WM tracts are also susceptible to the effects of healthy ageing, characterized by volume reduction.^52,53^ Although increased brain age is strongly associated with reductions in GM and WM volumes, our VBM analysis extends current understanding by identifying specific brain regions that may disproportionately contribute to accelerated brain ageing following stroke.

A key benefit of VLSM is its ability to identify all brain regions linked to variations in behavioural (or other continuous) scores, without being limited to predefined regions of interest.^20,54^ Here, VLSM applied to BAGCH revealed associations between the localization of the primary lesion and BAGCH: lesions in the striatocapsular brain region, including WM areas such as internal capsule (anterior and posterior limbs, retro-lenticular part), superior and posterior corona radiata, and the sagittal striatum, were related to high BAGCH, i.e. in patients with high BAGCH. This is noteworthy since injury to corticostriatal and thalamocortical projections has been shown to induce thalamic atrophy.^55,56^ Furthermore, thalamic atrophy, reported in normal ageing,^51^ is further accelerated in stroke,^57^ consistent with the results of our VBM analysis.

Some cross-sectional brain age studies in stroke have identified a correlation between a greater BAG and poorer motor outcome among patients.^2,23^ However, cross-sectional approaches may confound stroke-related brain age estimation with age-related neuronal decline or other pre-existing factors acting on degenerative processes.^58-61^ A previous study has therefore advocated the use of longitudinal imaging data to elucidate the temporal dynamics of brain age change.^8^ In our longitudinal study, we found that BAGCH was related to poor sensorimotor outcome at follow-up. Specifically, a 1-year increase in BAGCH was associated with a 6-unit decrease in Fugl–Meyer scores. Similar relationships were observed between BAGCH and mGS and manual dexterity, where increased BAGCH resulted in decreased strength and dexterity scores. In a previous longitudinal study, a significant relation was identified between proportional brain age difference and Barthel index,^9^ a measure of activities of daily living, not a sensorimotor outcome. To the best of our knowledge, this study is the first to identify a relationship between longitudinal brain age change and sensorimotor outcome post-stroke.

Chronological age is a strong risk factor for ischaemic stroke^62^ and an important biomarker for predicting upper limb function after stroke.^63^ However, this does not consider the phenomenon of accelerated post-stroke brain aging,^7,9^ which affects post-stroke functional outcome.^2,4,17,23^ Unlike chronological age, the concept of brain age captures the cumulative effects of genetics, lifestyle and disease on brain structure and function.^8,64^ Importantly, individuals with the same chronological age can have different brain ages due to varying levels of neurodegeneration, vascular health and exposure to risk factors such as hypertension or smoking. Furthermore, brain age serves as a better indicator of brain reserve: the brain's capacity to withstand ischaemic injury and adapt to damage, which significantly influences recovery outcome.^65^

Predicting motor outcome is of clinical relevance and a recent study showed that baseline motor function explains up to 70% of variance of motor outcome at 6 months.^29^ Similarly, weighted corticospinal tract lesion load is a further predictor and has been associated with worse motor outcome post-stroke.^66^ We found that in the absence of baseline motor function, which has been shown to explain up to two-thirds of the variance of motor outcome at 6 months post-stroke, BAGCH was significant in predicting motor outcome at follow-up. However, this effect became nonsignificant when baseline motor function scores were included in the regression model. Thus the role of baseline motor function predicting outcome far exceeds that of BAGCH. That notwithstanding, we found a significant relationship between weighted corticospinal tract lesion load and BAGCH. This relationship may suggest the presence of co-variance between BAGCH and baseline motor score, which may reflect a structural relation between greater corticospinal tract lesion load (mirrored in worse baseline motor function) and increased BAGCH. This is consistent with our VLSM results showing that high BAGCH was predicted by lesions to corticospinal tract and other pathways (thalamocortical projections, internal capsule and corona radiata) that have previously been related to poor baseline motor scores and motor recovery.^67,68^ Also, given that chronological age is used in predicting upper limb function after stroke,^63^ the complementary use of BAGCH as a predictor may provide a more personalized assessment based on a mechanistic understanding of stroke-induced brain ageing.

The presence of perilesional and distant brain changes due to post-stroke neuronal degeneration has been thoroughly documented in animal^69^ as well as in human studies.^12,70^ These changes exhibit distinct patterns dependent on lesion site, and they strongly impact the potential for motor recovery.^71^ Despite robust correlations between neuronal degeneration captured in VBM analysis and brain age, assessing brain age offers a distinct advantage in that it can be readily calculated based on clinical standard MRI and is indicative of the cumulative and diffuse impact of aging and health conditions over time, the latter driven by systemic processes such as inflammation, oxidative stress and neurodegeneration.

## Limitations

Several limitations need to be considered. First, this study lacked data on pre-existing comorbidities. Previous studies have found that pre-existing conditions, such as hypertension,^72^ diabetes^73^ and coronary artery disease,^2^ are also related to high brain age. Therefore, the brain age of our stroke patients may in part have been biased (increased) by these pre-existing conditions prior to their stroke. However, the use of longitudinal data and of relative measures, e.g. change of BAG, helps mitigate this problem. Second, 114 stroke patients were included, fewer than in other similar studies.^2,23^ However, we provide the first longitudinal data on brain ageing post-stroke in moderate to severe stroke as opposed to cross-sectional approaches previously used for assessing motor outcome in relation to brain age. The cross-sectional approach is most likely more strongly biased by other factors such as comorbidities, early life exposures and genetics.^8^ Third, WMH segmentation was performed using only T_1_-weighted images, rather than fluid-attenuated inversion recovery (FLAIR) imaging, considered the gold standard. This may have limited the sensitivity and specificity for identifying (classifying) WMHs, with a potential bias to underestimation. However, in a subset of 10 patients who had FLAIR images, we compared WMH segmentation between their T_1_-weighted only and their T_1_-weighted + FLAIR images: T_1_-weighted images tended to underestimate WMHs, but they showed a high correlation (*r* = 0.97, *P* < 0.001) with T_1_-weighted + FLAIR results. Finally, the metrics utilized to assess motor performance exhibited variability across the various study cohorts. This discrepancy has the potential to introduce bias into the regression analysis. To address this, a linear mixed regression model was employed, with the study cohort designated as a random effect to mitigate the impact of these variations and to ensure robustness of statistical analyses.

## Conclusion

To conclude, we show that stroke induces increased brain ageing during the first months post-stroke in patients with larger lesions. This increase in brain age is a result of the reduction in GM and WM volume (neurodegeneration) in sensory, motor and higher order cortical areas, as well as in subcortical structures. This study also provides evidence that BAGCH, which assesses the longitudinal change in brain age in stroke patients, can predict upper limb post-stroke motor outcome. Brain age can be readily calculated at the individual level using T_1_-weighted MRI and may be an important biomarker to consider for patient stratification and prediction of post-stroke outcome in future clinical trials.

## Supplementary Material

fcaf299_Supplementary_Data

## Data Availability

Anonymized data from this study is available, upon reasonable request, from the corresponding author and if it does not violate GDPR and local ethics committee rules. The codes used in this study are available in the Supplementary material.
