# Supplementary material for: Longitudinal brain ageing after stroke: a marker for neurodegeneration and its relevance for upper limb motor outcome
Source: Brain Commun. 2025 Aug 14;7(5):fcaf299. doi: 10.1093/braincomms/fcaf299 (PMC12399367; doi:10.1093/braincomms/fcaf299)
Supplement: fcaf299_Supplementary_Data [file fcaf299_supplementary_data.docx]

**Supplementary Table 1 | Voxel based morphometry analysis of areas of *white matter* volume reduction in the patient group with high change in brain age gap**

The high BAGCH group concerns N=57 patients. All t-values at *P_FWE_* <0.05.

| **Location** | **No. of voxels** | ***t*-value** |
| --- | --- | --- |
| Body of corpus callosum | 273 | 6.29 |
| Splenium of corpus callosum | 217 | 5.48 |
| Cerebral peduncle | 156 | 7.00 |
| Posterior limb of internal capsule | 337 | 7.21 |
| Retrolenticular part of internal capsule | 200 | 6.46 |
| Superior corona radiata | 891 | 7.31 |
| Posterior corona radiata | 332 | 6.05 |

**Supplementary Table 2 | Voxel based morphometry analysis of areas of *grey matter* volume reduction from baseline compared to follow-up**

| **Location (Broadman area)** | **No. of voxels** | **T value** | **MNI coordinates {mm}** | | |
| --- | --- | --- | --- | --- | --- |
|  |  |  | x | y | z |
| Right Thalamus | 10440 | 9.86 | 10 | -30 | 9 |
| Right Insula | 3475 | 8.40 | 45 | -8 | 3 |
| Right Precuneus | 3166 | 7.36 | 2 | -63 | 46 |
| Right Superior Frontal Gyrus | 228 | 7.31 | 28 | -9 | 66 |
| Right Precentral Gyrus | 389 | 6.89 | 50 | 8 | 38 |
| Right Middle Temporal Gyrus (BA21) | 646 | 6.55 | 57 | -34 | 0 |
| Right Middle Temporal Gyrus (BA19) | 154 | 6.12 | 54 | -60 | 16 |
| Right Cuneus | 340 | 6.00 | 12 | -76 | 33 |

All t-values at *P_FWE_* <0.05.

**Supplementary Table 3 | Voxel based morphometry analysis of areas of *white matter* volume reduction from baseline compared to follow-up**

| **Location** | **No. of voxels** | ***t*-value** |
| --- | --- | --- |
| Middle cerebellar peduncle | 9082 | 9.37 |
| Pontine_crossing_tract_(a_part_of_MCP) | 1270 | 8.56 |
| Genu_of_corpus_callosum | 1025 | 7.30 |
| Body_of_corpus_callosum | 5181 | 8.87 |
| Splenium_of_corpus_callosum | 1886 | 7.38 |
| Corticospinal_tract_L | 543 | 7.76 |
| Corticospinal_tract_R | 1370 | 8.61 |
| Inferior_cerebellar_peduncle_L | 220 | 7.32 |
| Superior_cerebellar_peduncle_L | 254 | 7.36 |
| Superior_cerebellar_peduncle_R | 266 | 7.80 |
| Anterior_limb_of_internal_capsule_R | 772 | 6.64 |
| Posterior_limb_of_internal_capsule_R | 135 | 6.66 |
| Retrolenticular_part_of_internal_capsule_R | 1503 | 7.58 |
| Anterior_corona_radiata_R | 4778 | 8.13 |
| Superior_corona_radiata_R | 5630 | 9.53 |
| Posterior_corona_radiata_R | 1916 | 8.82 |
| Posterior_thalamic_radiation_(include_optic_radiation)_R | 850 | 6.56 |
| Sagittal_stratum_R | 542 | 7.15 |
| External_capsule_L | 156 | 5.82 |
| Cingulum_(cingulate_gyrus)_R | 631 | 6.73 |
| Fornix_cres/Stria_terminalis_R | 801 | 8.08 |
| Superior_longitudinal_fasciculus_R | 426 | 7.82 |

R: right, L: left

All t-values at *P_FWE_* <0.05.

**Supplementary Table 4 | Voxel-based lesion symptom mapping applied to brain age: lesioned white matter areas associated with high change in brain age gap**

| **Location** | **No. of voxels** | ***t*-value** |
| --- | --- | --- |
| Anterior limb of internal capsule | 211 | 5.23 |
| Posterior limb of internal capsule | 593 | 5.14 |
| Retrolenticular part of internal capsule | 295 | 5.42 |
| Superior corona radiata | 138 | 4.80 |
| Posterior corona radiata | 10 | 4.52 |
| External capsule | 818 | 5.53 |
| Superior longitudinal fasciculus | 53 | 4.60 |
| Superior fronto-occipital fasciculus | 124 | 5.35 |

The high BAGCH group concerns N=57 patients. All t-values at *P_FWE_* <0.05.

**Supplementary Table 5 | Prediction of chronic sensorimotor post-stoke outcome**

| **Outcome variable** | **Predictor** | **β** | **SE** | **t value** | **p value** |
| --- | --- | --- | --- | --- | --- |
| **FMA_UE**  N = 86  R^2^ = 0.77 | BAGCH | -0.63 | 1.53 | -0.41 | 0.68 |
|  | Age | -1.57 | 1.73 | -0.91 | 0.37 |
|  | Sex | -1.03 | 1.51 | -0.68 | 0.50 |
|  | Days post-stroke | -2.16 | 1.36 | -1.59 | 0.12 |
|  | Days between scans | -4.58 | 1.91 | -2.39 | 0.02 |
|  | TIV | 0.84 | 1.78 | 0.47 | 0.64 |
|  | WMH | -1.80 | 1.47 | -1.23 | 0.22 |
|  | **Baseline FMA_UE** | **0.86** | **0.06** | **14.05** | **<0.001** |
|  | Lesion side | -0.57 | 1.37 | -0.42 | 0.68 |
|  | Baseline BAG | 0.39 | 1.54 | 0.25 | 0.80 |
| **mGS**  N = 114  R^2^ = 0.77 | BAGCH | -0.04 | 0.02 | -1.69 | 0.09 |
|  | Age | -0.03 | 0.02 | -1.19 | 0.24 |
|  | Sex | 0.01 | 0.02 | 0.30 | 0.77 |
|  | Days post-stroke | -0.01 | 0.02 | -0.45 | 0.66 |
|  | Days between scans | -0.01 | 0.02 | -0.38 | 0.71 |
|  | TIV | 0.002 | 0.03 | 0.10 | 0.92 |
|  | WMH | -0.01 | 0.02 | -0.23 | 0.82 |
|  | **Baseline mGS** | **0.85** | **0.05** | **15.88** | **<0.001** |
|  | Lesion side | -0.02 | 0.02 | -1.14 | 0.23 |
|  | Baseline BAG | -0.03 | 0.02 | 1.19 | 0.24 |
| **DextSc**  N = 71  R^2^ = 0.73 | BAGCH | -0.03 | 0.03 | -1.16 | 0.25 |
|  | Age | 6.8x10^-5^ | 0.03 | 0.02 | 0.98 |
|  | Sex | 0.01 | 0.03 | 0.33 | 0.74 |
|  | Days post-stroke | -0.04 | 0.04 | -1.06 | 0.29 |
|  | Days between scans | -0.02 | 0.07 | -0.32 | 0.75 |
|  | TIV | 0.03 | 0.03 | 0.82 | 0.42 |
|  | WMH | -0.03 | 0.02 | -1.08 | 0.28 |
|  | **Baseline DextSc** | **0.87** | **0.08** | **10.38** | **<0.001** |
|  | Lesion side | 0.02 | 0.03 | 0.67 | 0.51 |
|  | Baseline BAG | -0.02 | 0.03 | -0.73 | 0.47 |

BAGCH = brain age gap change; FMA-UE = Fugl-Meyer Assessment of upper extremity; mGS = maximum grip strength; DextSc = strength dexterity test (contralesional); TIV = total intracranial volume; WMH = white matter hyperintensities; Beta = standardized regression coefficient for each predictor variable. SE = standardized error. Significant predictors are shown in bold.

**Supplementary Figure 1: Correlation between brain age and chronological age at baseline.**


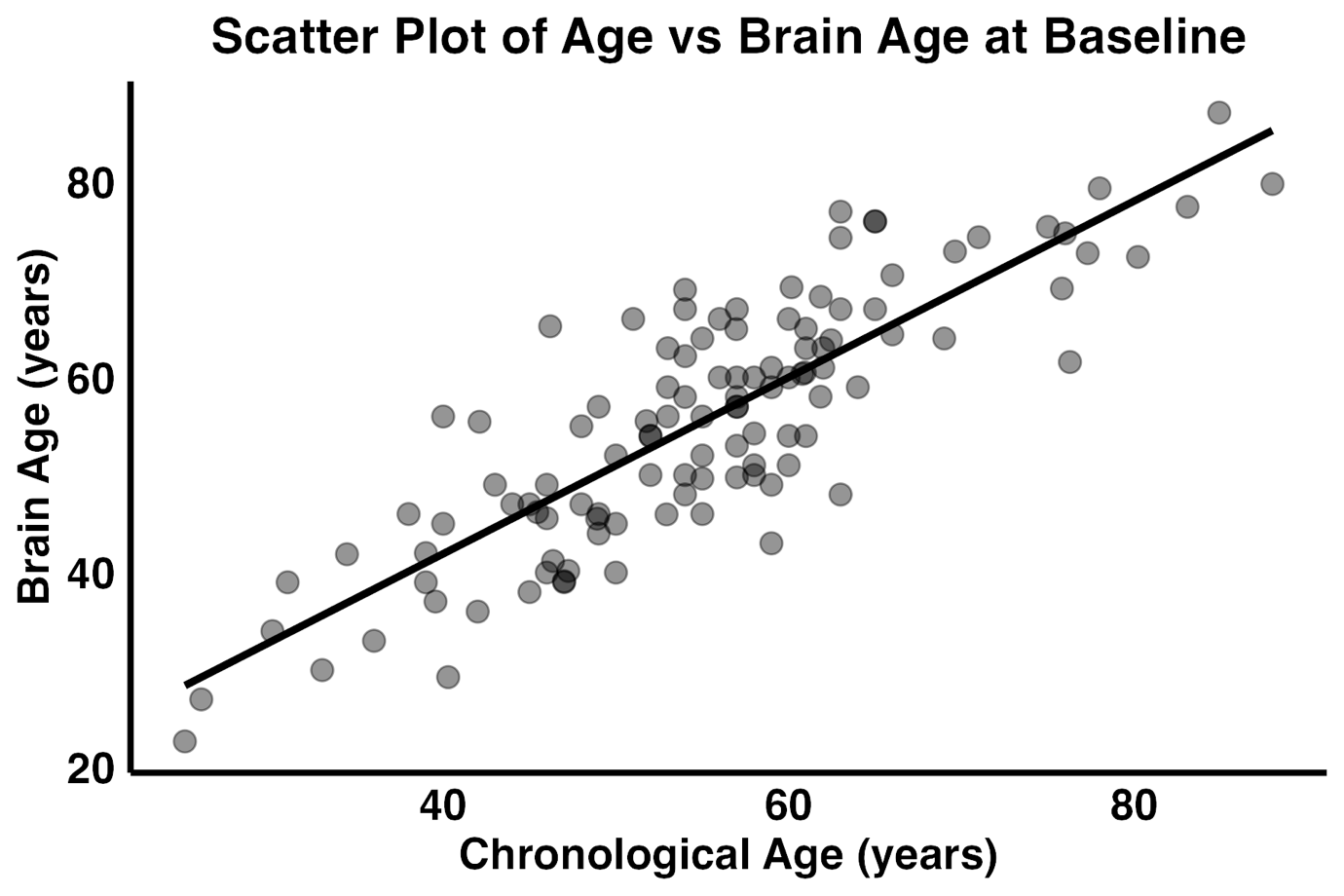


Scatter plot shows the correlation between chronological age and brain age at baseline. N=114, r=0.84, p<0.001.

**Supplementary Figure 2: Correlation between brain age and chronological age at follow-up.**


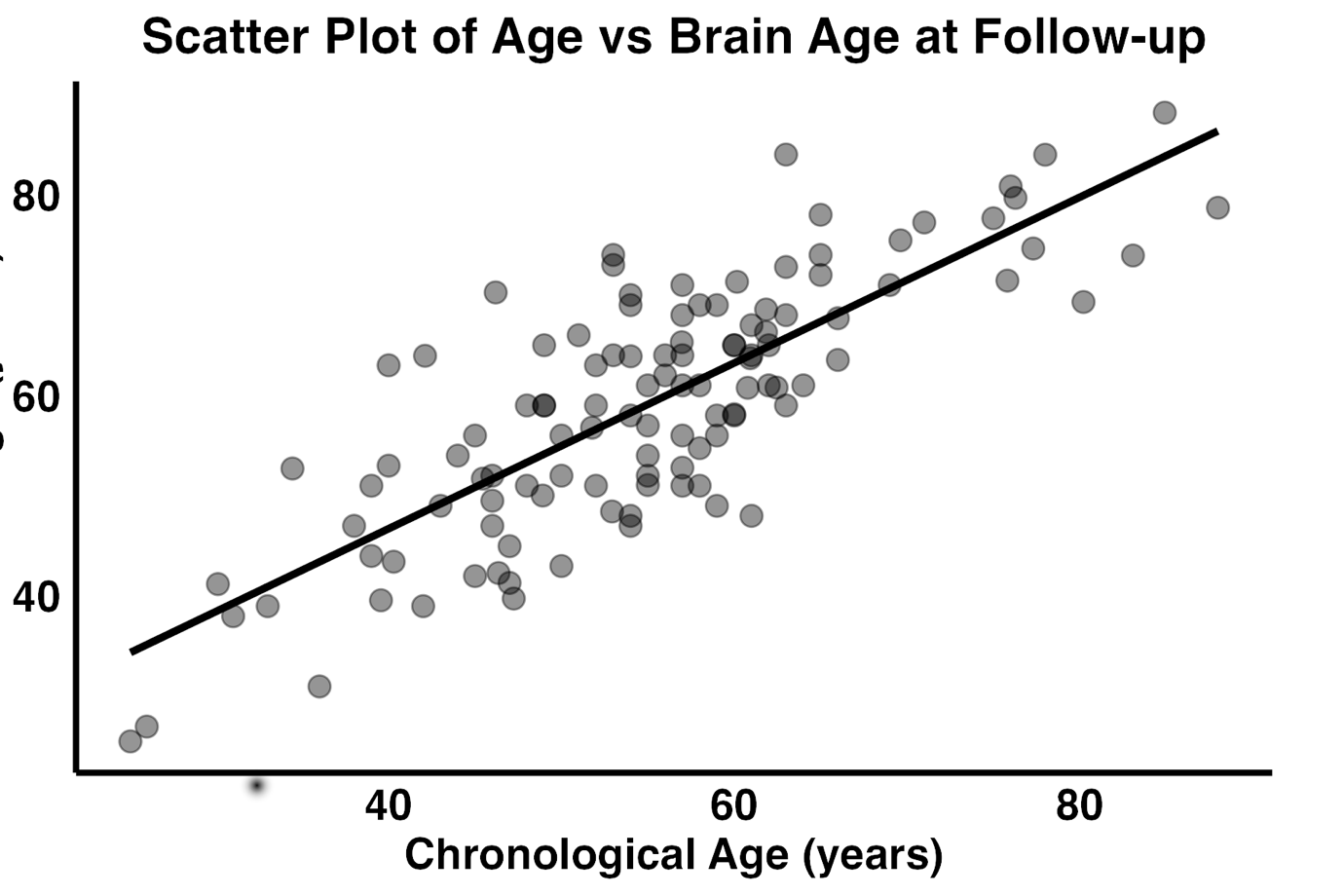


Scatter plot shows the correlation between chronological age and brain age at follow-up. N=114, r=0.80, p<0.001.

**Supplementary Figure 3: Distribution of BAG change**


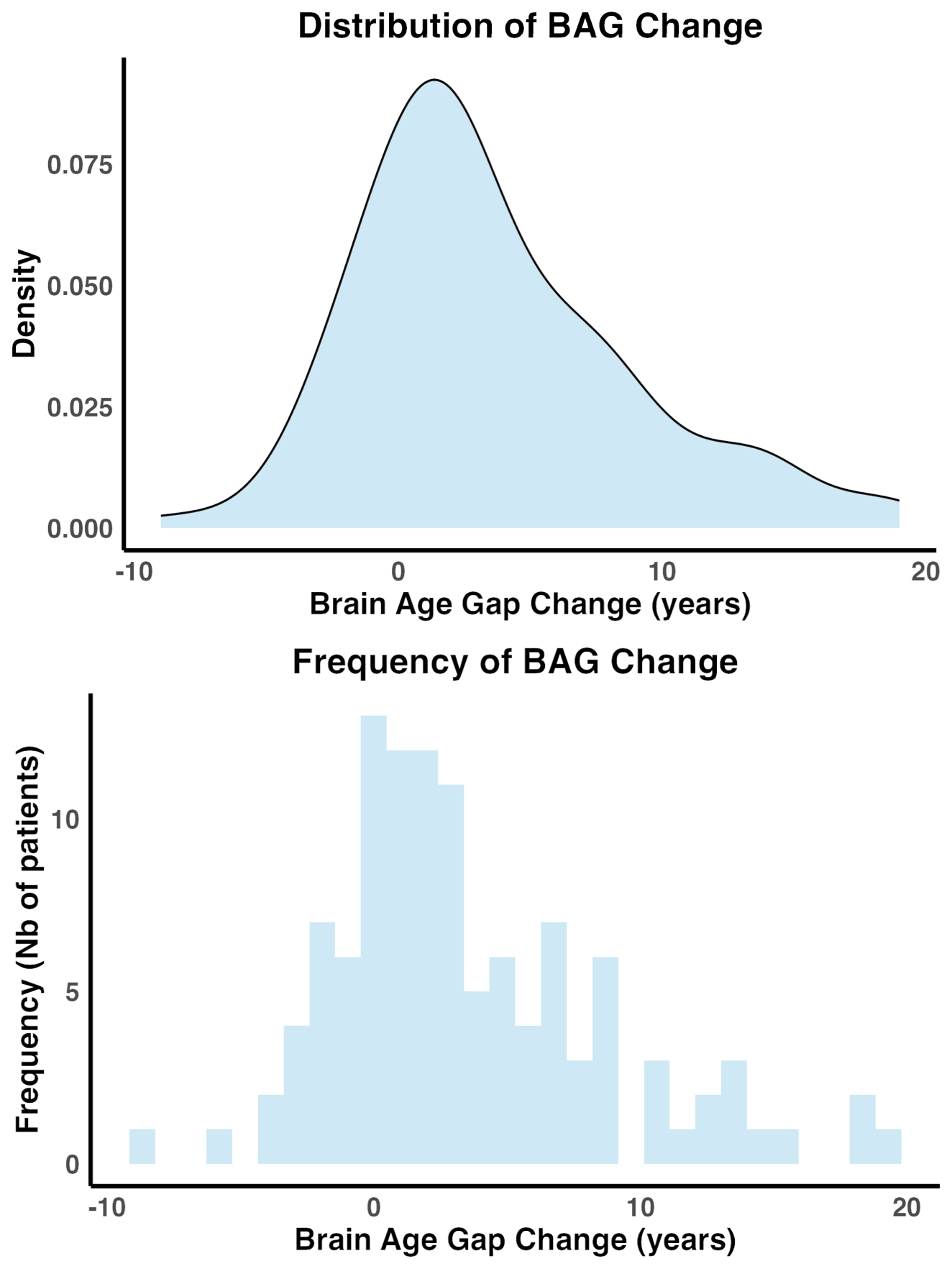


Above: Density plot showing the distribution of brain age gap (BAG) change.

Below: Frequency plot showing the distribution of brain age gap (BAG) change.

N = 114.

**Supplementary Figure 4: longitudinal BAG change for each participant**


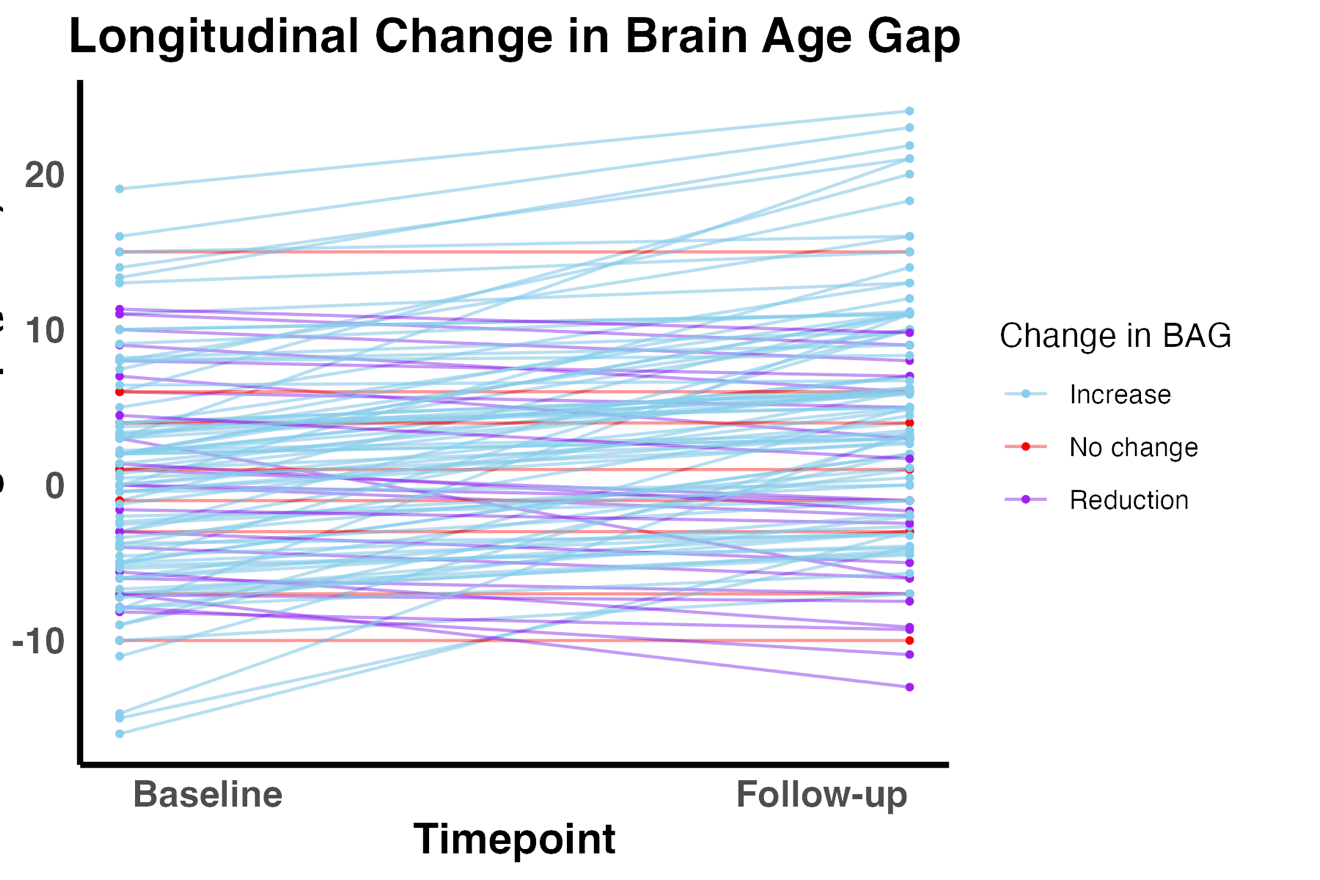


Line plot showing the change of individual BAG from baseline to follow-up. Color: light blue: participants who show an increase in BAG over time; red: participants who do not show any change over time (BAG at baseline equals the BAG at follow-up); purple: participants who show a reduction in BAG over time. N = 114

**Supplementary Figure 5: Voxel based morphometry analysis of areas of *grey/white matter* volume reduction at baseline and at follow-up**

**
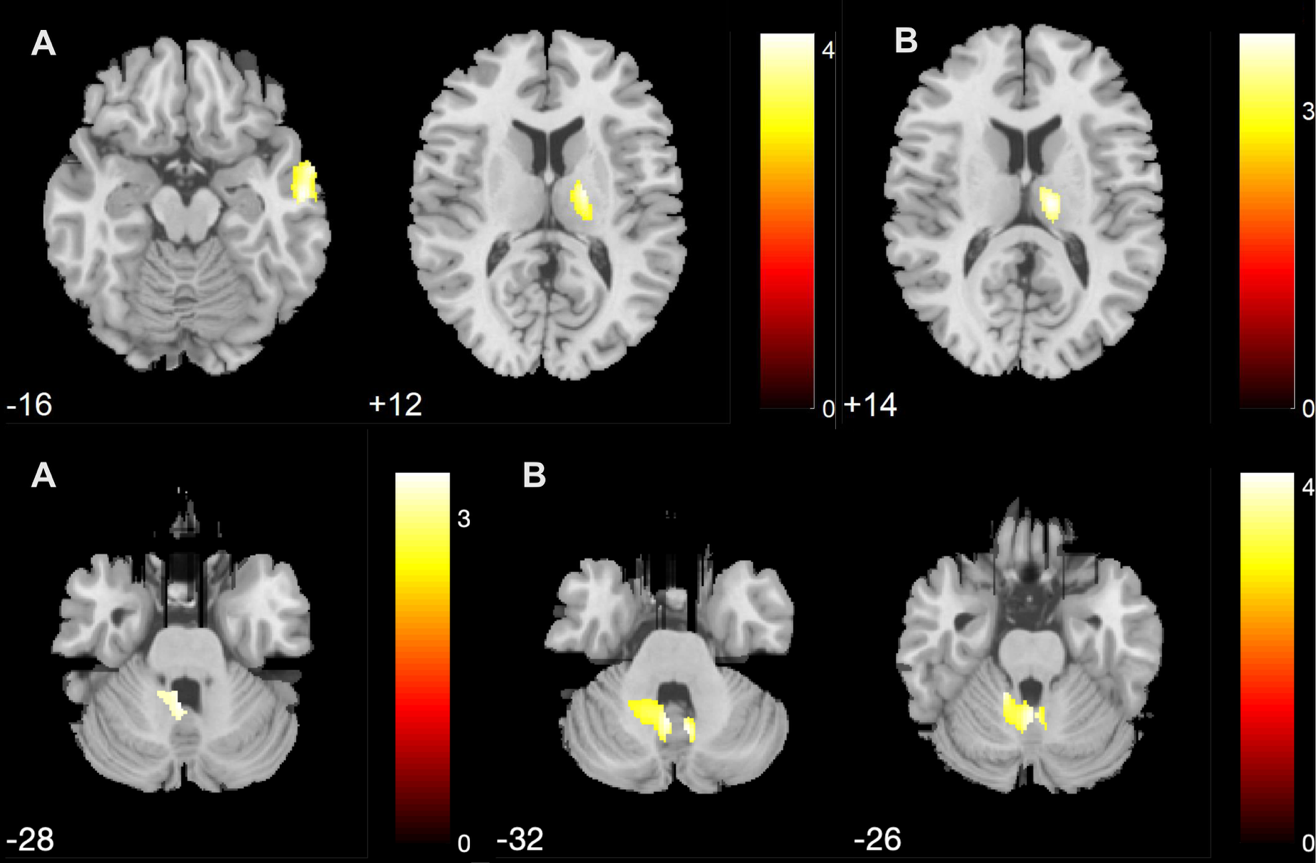
**

VBM analysis showing regions of decreased grey matter and white matter volume in patients at baseline and follow-up. Colour scale bar: magnitude of T values. Numbers correspond to the coordinate of the axial slice in the MNI space. All t-values at P*_UNCORR_* < 0.05; (N = 114).

Top row: A: Grey matter volume reduction at baseline; B: Grey matter volume reduction at follow-up.

Bottom row: A: White matter volume reduction at baseline; B: White matter volume reduction at follow-up.

**Supplementary Figure 6: Voxel based morphometry analysis of areas of *grey matter* volume reduction from baseline compared to follow-up**

**
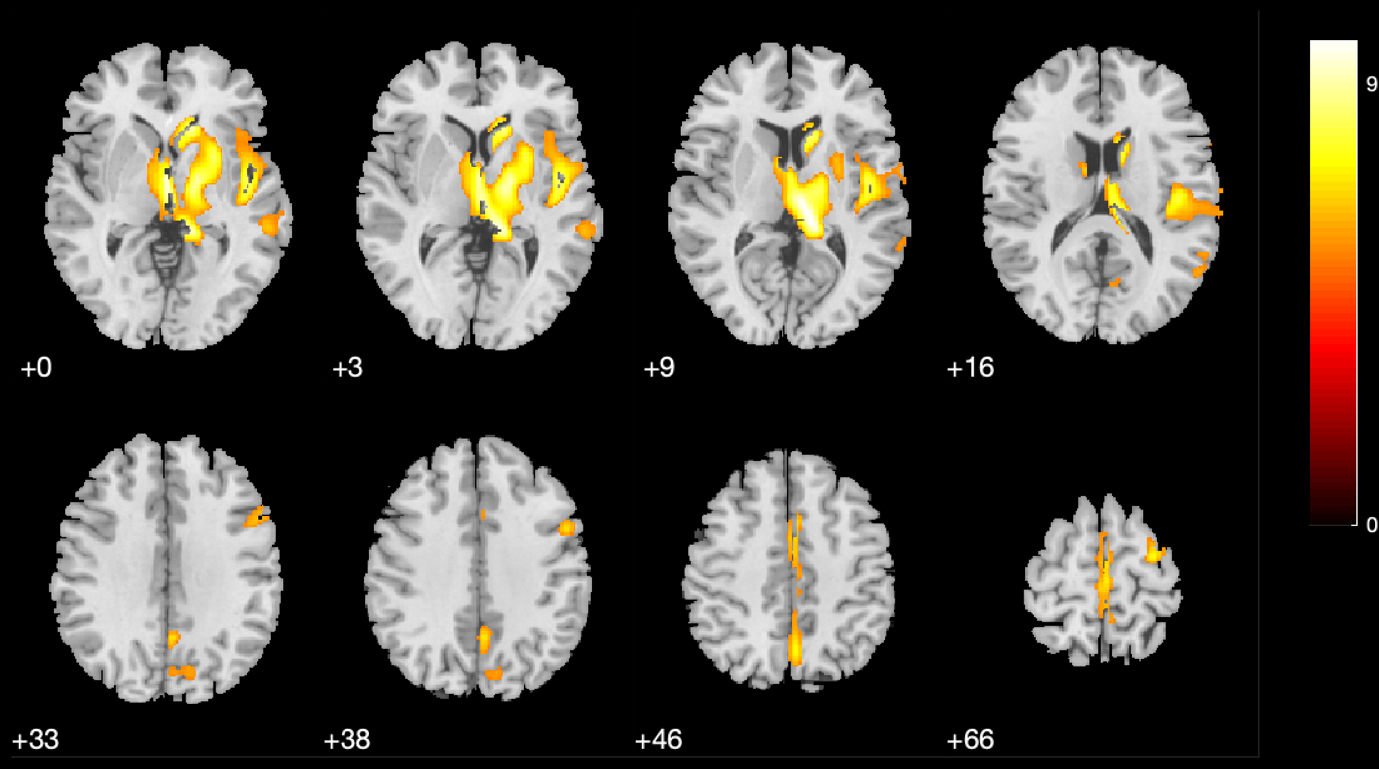
**

VBM analysis showing regions of decreased grey matter volume in patients over time (from baseline to follow-up). Colour scale: magnitude of T values. Numbers correspond to the coordinate of the axial slice in the MNI space. All t-values at P*_FWE_* < 0.05; (N = 114). See supplementary Table 2 for identification of affected regions.

**Supplementary Figure 7: Voxel based morphometry analysis of areas of *white matter* volume reduction from baseline compared to follow-up**


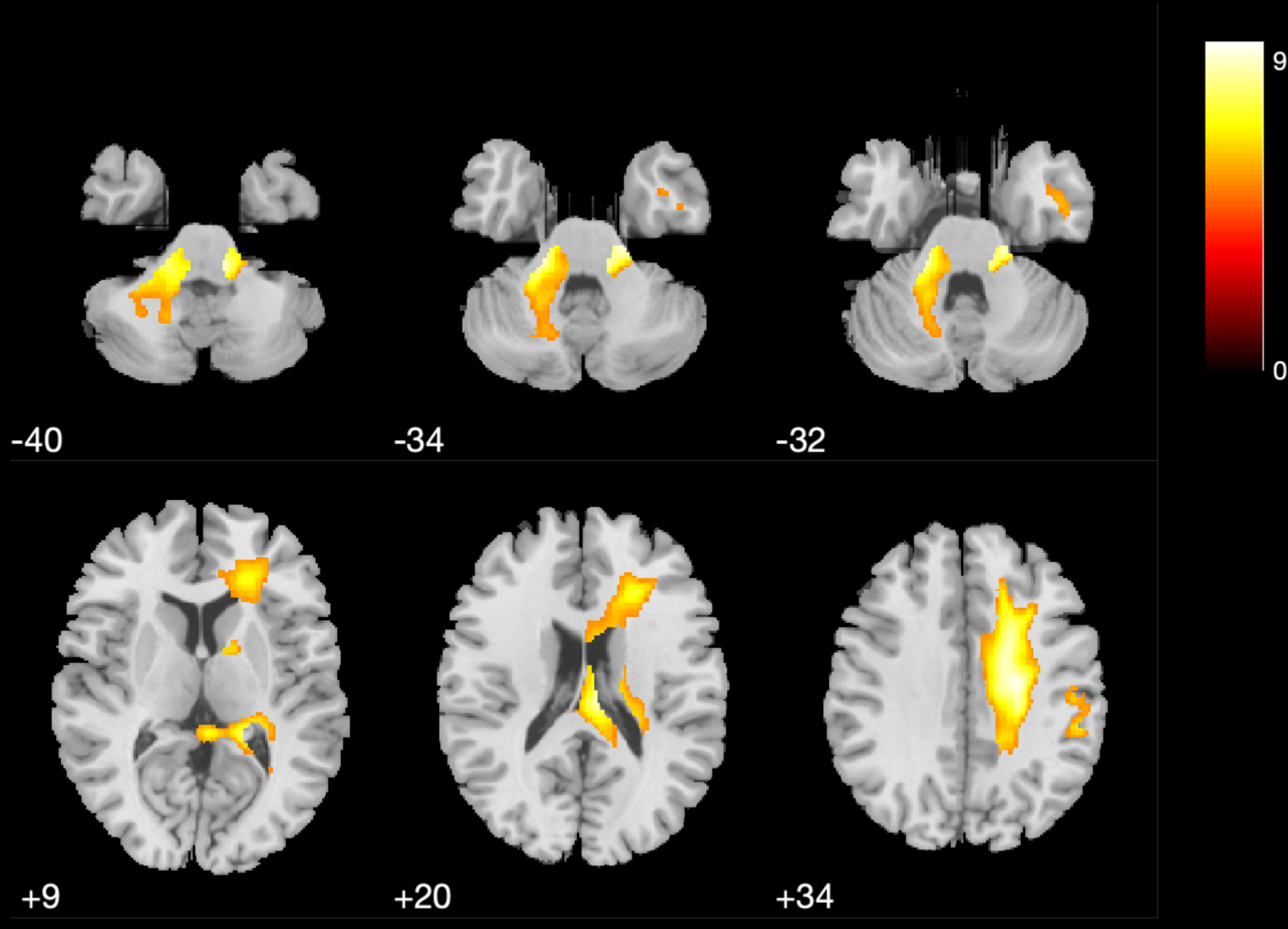


VBM analysis showing regions of decreased white matter volume in patients over time (from baseline to follow-up). Colour scale: magnitude of T values. Numbers correspond to the coordinate of the axial slice in the MNI space. All t-values at P*_FWE_* < 0.05; (N = 114). See supplementary Table 3 for identification of affected regions.

Code used in the manuscript

library(olsrr)

library(readxl)

library(writexl)

library(dplyr)

library(rstatix)

library(ggplot2)

library(tidyverse)

library(car)

library(rcompanion)

library(ggpubr)

library(boot)

library(scales)

library(ggsignif)

library(mediation)

library(flexplot)

library(lme4)

library(lmerTest)

library(ggplot2)

library(interactions)

data_1 = read_excel("/../../../data.xlsx")

data_1

summary(data_1)

# Create the scatter plot with regression line

scatter_plot <- ggplot(data_1, aes(x = AGE, y = BA_T1_LE)) +

geom_point(color = "black", size = 3, alpha = 0.7) + # Points with color and size

geom_smooth(method = "lm", se = FALSE, color = "black", linetype = "solid", size = 1.2) + # Regression line

labs(

title = "Scatter Plot of Age vs Brain Age at Baseline",

x = "Chronological Age (years)",

y = "Brain Age (years)"

) +

theme_minimal() + # Clean minimal theme

theme(

plot.title = element_text(hjust = 0.5, size = 16, face = "bold", color = "black"),

axis.title = element_text(size = 14, face = "bold", color = "black"),

axis.line = element_line(color = "black", size = 1), # Black and thicker axis line

axis.text = element_text(size = 14, face = "bold", color = "black"),

panel.grid = element_blank() # Remove grid lines

)

# Display the plot

print(scatter_plot)

ggsave(

filename = "scatter_plot_1.png", # or .tiff, .jpeg, etc.

plot = last_plot(), # or specify your plot object, e.g., plot = p

width = 6, # width in inches (adjust as needed)

height = 4, # height in inches (adjust as needed)

units = "in",

dpi = 300

)

#Distribution of Brain age gap at baseline, follow-up and BAGCH

ggplot(data_1, aes(x = BAG_CH)) +

geom_density(fill = "skyblue", alpha = 0.5) +

labs(

title = "Distribution of BAG Change",

x = "Brain Age Gap Change (years)",

y = "Density"

) +

theme_minimal() +

theme(

panel.grid = element_blank(), # Remove gridlines

axis.line = element_line(color = "black", size = 1), # Add black axis lines

axis.title = element_text(size = 14, face = "bold"),

axis.text = element_text(size = 12, face = "bold"),

plot.title = element_text(size = 16, face = "bold", hjust = 0.5)

)

ggplot(data_1, aes(x = BAG_CH)) +

geom_histogram(fill = "skyblue", alpha = 0.5, bins = 30) +

labs(

title = "Frequency of BAG Change",

x = "Brain Age Gap Change (years)",

y = "Frequency (Nb of patients)"

) +

theme_minimal() +

theme(

panel.grid = element_blank(),

axis.line = element_line(color = "black", size = 1),

axis.title = element_text(size = 14, face = "bold"),

axis.text = element_text(size = 12, face = "bold"),

plot.title = element_text(size = 16, face = "bold", hjust = 0.5)

)

ggsave(

filename = "brain_age_gap_density_32.png", # or .tiff, .jpeg, etc.

plot = last_plot(), # or specify your plot object, e.g., plot = p

width = 6, # width in inches (adjust as needed)

height = 4, # height in inches (adjust as needed)

units = "in",

dpi = 300

)

# Reshape data to long format

data_long <- pivot_longer(

data_1,

cols = c(BAG_3W_LE, BAG_6M_LE),

names_to = "Timepoint",

values_to = "BAG"

)

# Map timepoints to custom labels

data_long$Timepoint <- factor(

data_long$Timepoint,

levels = c("BAG_3W_LE", "BAG_6M_LE"),

labels = c("baseline", "follow up")

)

# Define custom colors

fill_colors <- c("baseline" = "skyblue", "follow up" = "lightgreen")

line_colors <- c("baseline" = "blue", "follow up" = "green") # deep blue and green

# Calculate group means

means <- data_long %>%

group_by(Timepoint) %>%

summarise(mean_BAG = mean(BAG, na.rm = TRUE))

# Plot with enhanced dashed mean lines

ggplot(data_long, aes(x = BAG, fill = Timepoint)) +

geom_density(aes(color = Timepoint), alpha = 0.5) +

geom_vline(data = means, aes(xintercept = mean_BAG, color = Timepoint),

linetype = "dashed", size = 1.2) +

scale_fill_manual(values = fill_colors, name = "Timepoint") +

scale_color_manual(values = line_colors, name = "Timepoint") +

labs(

title = "A. Distribution of BAG at Baseline and Follow-up",

x = "Brain Age Gap (years)",

y = "Density"

) +

theme_minimal() +

theme(

panel.grid = element_blank(),

axis.line = element_line(color = "black", size = 1),

axis.title = element_text(size = 14, face = "bold"),

axis.text = element_text(size = 12, face = "bold"),

plot.title = element_text(size = 16, face = "bold", hjust = 0.5)

)

ggsave(

filename = "brain_age_gap_density_new.tiff", # or .tiff, .jpeg, etc.

plot = last_plot(), # or specify your plot object, e.g., plot = p

width = 6, # width in inches (adjust as needed)

height = 4, # height in inches (adjust as needed)

units = "in",

dpi = 300

)

#Distribution of Brain age gap at baseline, follow-up showing on one plot

# Reshape data to long format

data_long <- pivot_longer(

data_1,

cols = c(BAG_3W_LE, BAG_6M_LE),

names_to = "Timepoint",

values_to = "BAG"

)

# Map your timepoints to custom labels

data_long$Timepoint <- factor(

data_long$Timepoint,

levels = c("BAG_3W_LE", "BAG_6M_LE"),

labels = c("baseline", "follow up")

)

# Define your custom colors

custom_colors <- c("baseline" = "skyblue", "follow up" = "lightgreen")

# Plot

ggplot(data_long, aes(x = BAG, fill = Timepoint, color = Timepoint)) +

geom_density(alpha = 0.5) +

scale_fill_manual(values = custom_colors, name = "Timepoint") +

scale_color_manual(values = custom_colors, name = "Timepoint") +

labs(

title = "Distribution of BAG at Baseline and Follow up",

x = "Brain Age Gap (years)",

y = "Density"

) +

theme_minimal() +

theme(

panel.grid = element_blank(),

axis.line = element_line(color = "black", size = 1),

axis.title = element_text(size = 14, face = "bold"),

axis.text = element_text(size = 12, face = "bold"),

plot.title = element_text(size = 16, face = "bold", hjust = 0.5)

)

ggplot(data_long, aes(x = BAG, fill = Timepoint, color = Timepoint)) +

geom_histogram(

position = "identity",

alpha = 0.5,

bins = 30

) +

scale_fill_manual(values = custom_colors, name = "Timepoint") +

scale_color_manual(values = custom_colors, name = "Timepoint") +

labs(

title = "B. Frequency of BAG at Baseline and Follow up",

x = "Brain Age Gap (years)",

y = "Frequency (Nb of Patients)"

) +

theme_minimal() +

theme(

panel.grid = element_blank(),

axis.line = element_line(color = "black", size = 1),

axis.title = element_text(size = 14, face = "bold"),

axis.text = element_text(size = 12, face = "bold"),

plot.title = element_text(size = 16, face = "bold", hjust = 0.5)

)

ggsave(

filename = "brain_age_gap_freq.png", # or .tiff, .jpeg, etc.

plot = last_plot(), # or specify your plot object, e.g., plot = p

width = 6, # width in inches (adjust as needed)

height = 4, # height in inches (adjust as needed)

units = "in",

dpi = 300

)

#Line plot mapping BAG at baseline to follow-up for each subject

# Step 1: Calculate change category for each subject

data_1 <- data_1 %>%

mutate(

Change = case_when(

BAG_6M_LE > BAG_3W_LE ~ "Increase",

BAG_6M_LE < BAG_3W_LE ~ "Reduction",

TRUE ~ "No change"

)

)

# Step 2: Reshape to long format for plotting

data_long <- data_1 %>%

dplyr::select(Subject, BAG_3W_LE, BAG_6M_LE, Change) %>%

pivot_longer(

cols = c(BAG_3W_LE, BAG_6M_LE),

names_to = "Timepoint",

values_to = "BAG"

)

#Step 3: Rename

data_long$Timepoint <- factor(

data_long$Timepoint,

levels = c("BAG_3W_LE", "BAG_6M_LE"),

labels = c("Baseline", "Follow-up")

)

data_long <- data_long %>%

mutate(

Timepoint_num = ifelse(Timepoint == "Baseline", 1, 10)

)

# Step 4: Plot

ggplot(data_long, aes(x = Timepoint_num, y = BAG, group = Subject, color = Change)) +

geom_line(size = 0.5, alpha = 0.7) +

geom_point(size = 0.7) +

scale_x_continuous(

breaks = c(2, 9),

labels = c("Baseline", "Follow-up")

) +

scale_color_manual(

values = c("Increase" = "skyblue", "Reduction" = "purple", "No change" = "red"),

name = "Change in BAG"

) +

labs(

title = "Longitudinal Change in Brain Age Gap",

x = "Timepoint",

y = "Brain Age Gap (years)"

) +

theme_minimal() +

theme(

panel.grid = element_blank(),

axis.line = element_line(color = "black", size = 1),

axis.title = element_text(size = 14, face = "bold"),

axis.text = element_text(size = 12, face = "bold"),

plot.title = element_text(size = 16, face = "bold", hjust = 0.5)

)

ggsave(

filename = "brain_age_gap_change_LINE.png", # or .tiff, .jpeg, etc.

plot = last_plot(), # or specify your plot object, e.g., plot = p

width = 6, # width in inches (adjust as needed)

height = 4, # height in inches (adjust as needed)

units = "in",

dpi = 300

)

n = sum(data_1$BAG_CH == 0)

# Calculate median BAG

median_bag_ch = median(data_1$BAG_CH, na.rm = TRUE)

# Count number above the median

n_above <- sum(data_1$BAG_CH > median_bag_ch, na.rm = TRUE)

# Count number below the median

n_below <- sum(data_1$BAG_CH <= median_bag_ch, na.rm = TRUE)

# Create median split variable

# Comparing the two groups if they are similar in age, les vol, etc

data_1$BAG_CH_group = ifelse(data_1$BAG_CH <= median_bag, "Low", "High")

# Perform t-test

result = t.test(AGE ~ BAG_CH_group, data = data_1)

print(result)

result = t.test(les_vol_3w ~ BAG_CH_group, data = data_1)

print(result)

result = t.test(FMA_1 ~ BAG_CH_group, data = data_1)

print(result)

result = t.test(mGS_1 ~ BAG_CH_group, data = data_1)

print(result)

result = t.test(DextSc_1 ~ BAG_CH_group, data = data_1)

print(result)

result = t.test(wCSTLL_1 ~ BAG_CH_group, data = data_1)

print(result)

# Calculate means and do a paired t-test

mean(data_1$BAG_3W_LE)

mean(data_1$BAG_6M_LE)

t_test_result <- t.test(data_1$BAG_3W_LE, data_1$BAG_6M_LE, paired = TRUE)

print(t_test_result)

# Convert data to long format

data_long <- data.frame(

TimePoint = rep(c("Baseline", "Follow-up"), each = nrow(data_1)),

BAG = c(data_1$BAG_3W_LE, data_1$BAG_6M_LE)

)

# Make sure TimePoint is a factor with consistent order

data_long$TimePoint <- factor(data_long$TimePoint, levels = c("Baseline", "Follow-up"))

plot <- ggplot(data_long, aes(x = TimePoint, y = BAG, fill = TimePoint)) +

geom_violin(trim = FALSE, alpha = 0.6, color = NA) + # Violin plot

geom_boxplot(width = 0.1, outlier.shape = NA, alpha = 0.5, color = "black", size = 0.5) + # Boxplot overlay

geom_jitter(width = 0.1, size = 1, alpha = 0.6, color = "black") + # Data points

stat_summary(fun = mean, geom = "point", shape = 18, size = 4, color = "red") + # Mean point

scale_fill_manual(values = c("Baseline" = "skyblue", "Follow-up" = "lightgreen")) +

theme_minimal() +

labs(

title = "Comparison of BAG at baseline and follow-up",

x = "Time after stroke",

y = "BAG (years)"

) +

theme(

plot.title = element_text(hjust = 0.5, size = 16, face = "bold", color = "black"),

axis.title = element_text(size = 14, face = "bold", color = "black"),

axis.line = element_line(color = "black", size = 1),

axis.text = element_text(size = 14, face = "bold", color = "black"),

legend.position = "none",

panel.grid = element_blank()

)

# Show the plot

print(plot)

ggsave(

filename = "t-test.png", # or .tiff, .jpeg, etc.

plot = last_plot(), # or specify your plot object, e.g., plot = p

width = 6, # width in inches (adjust as needed)

height = 4, # height in inches (adjust as needed)

units = "in",

dpi = 300

)

#linear mixed effect model

# Rescale the predictor variables (e.g., BAG_CH, AGE, AGE_SQ, SEX, DAYS_POST_STROKE, DAYS_BTN_SCANS)

data_1$BAG_CH_scaled = scale(data_1$BAG_CH)

data_1$AGE_scaled = scale(data_1$AGE)

data_1$side_scaled = scale(data_1$side)

data_1$SEX_scaled = scale(data_1$SEX)

data_1$DAYS_POST_STROKE_scaled = scale(data_1$DAYS_POST_STROKE)

data_1$DAYS_BTN_SCANS_scaled = scale(data_1$DAYS_BTN_SCANS)

data_1$TIV_scaled = scale(data_1$TIV)

data_1$WMH_T1_scaled = scale(data_1$WMH_T1)

data_1$BAG_3W_LE_scaled = scale(data_1$BAG_3W_LE)

# Fit the model with the rescaled variables

#(FMA_Outcome)

model_scaled_FMA = lmer(FMA_2 ~ BAG_CH_scaled + AGE_scaled + SEX_scaled +

DAYS_POST_STROKE_scaled + DAYS_BTN_SCANS_scaled + TIV_scaled + WMH_T1_scaled + BAG_3W_LE_scaled + side_scaled + (1 | COHORT),

data = data_1)

summary(model_scaled_FMA)

# Fit the model with the rescaled variables (mGS_Outcome)

model_scaled_mGS = lmer(mGS_2 ~ BAG_CH_scaled + AGE_scaled + SEX_scaled +

DAYS_POST_STROKE_scaled + DAYS_BTN_SCANS_scaled + TIV_scaled + WMH_T1_scaled + BAG_3W_LE_scaled + side_scaled + (1 | COHORT),

data = data_1)

summary(model_scaled_mGS)

# Fit the model with the rescaled variables (DextSc_Outcome)

model_scaled_DextSc = lmer(DextSc_2 ~ BAG_CH_scaled + AGE_scaled + SEX_scaled +

DAYS_POST_STROKE_scaled + DAYS_BTN_SCANS_scaled + TIV_scaled + WMH_T1_scaled + BAG_3W_LE_scaled + side_scaled + (1 | COHORT),

data = data_1)

summary(model_scaled_DextSc)

# Fit the model with the rescaled variables with baseline motor score as covariate

#(FMA_Outcome)

model_scaled_FMA = lmer(FMA_2 ~ FMA_1 + BAG_CH_scaled + AGE_scaled + SEX_scaled +

DAYS_POST_STROKE_scaled + DAYS_BTN_SCANS_scaled + TIV_scaled + WMH_T1_scaled + BAG_3W_LE_scaled + side_scaled + (1 | COHORT),

data = data_1)

summary(model_scaled_FMA)

# Fit the model with the rescaled variables (mGS_Outcome)

model_scaled_mGS = lmer(mGS_2 ~ mGS_1 + BAG_CH_scaled + AGE_scaled + SEX_scaled +

DAYS_POST_STROKE_scaled + DAYS_BTN_SCANS_scaled + TIV_scaled + WMH_T1_scaled + BAG_3W_LE_scaled + side_scaled + (1 | COHORT),

data = data_1)

summary(model_scaled_mGS)

# Fit the model with the rescaled variables (DextSc_Outcome)

model_scaled_DextSc = lmer(DextSc_2 ~ DextSc_1 + BAG_CH_scaled + AGE_scaled + SEX_scaled +

DAYS_POST_STROKE_scaled + DAYS_BTN_SCANS_scaled + TIV_scaled + WMH_T1_scaled + BAG_3W_LE_scaled + side_scaled + (1 | COHORT),

data = data_1)

summary(model_scaled_DextSc)

#Paired t-test between BAG at baseline and follow-up

# Calculate means

mean_3W <- mean(data_1$BAG_3W_LE)

mean_6M <- mean(data_1$BAG_6M_LE)

cat("Mean of BAG_3W_LE:", mean_3W, "\n")

cat("Mean of BAG_6M_LE:", mean_6M, "\n")

# Convert data to long format

data_long <- data.frame(

TimePoint = rep(c("3 Weeks", "6 Months"), each = nrow(data_1)),

BAG = c(data_1$BAG_3W_LE, data_1$BAG_6M_LE)

)

# Create the boxplot with significance bar

plot <- ggplot(data_long, aes(x = TimePoint, y = BAG, fill = TimePoint)) +

geom_boxplot(width = 0.5, outlier.shape = NA, alpha = 0.7) + # Boxplot with no outliers

theme_minimal() +

labs(

title = "Comparison of BAG at 3 Weeks and 6 Months",

x = "Time Point",

y = "BAG"

) +

scale_fill_manual(values = c("skyblue", "lightgreen")) +

theme(

plot.title = element_text(hjust = 0.5, size = 16, face = "bold", color = "black"),

axis.title = element_text(size = 14, face = "bold", color = "black"),

axis.text = element_text(size = 14, face = "bold", color = "black"),

legend.position = "none"

) +

geom_signif(

comparisons = list(c("3 Weeks", "6 Months")), # Specify groups to compare

map_signif_level = TRUE, # Automatically adds significance level

y_position = max(data_long$BAG) + 3, # Position of the bar above the boxes

textsize = 5

)

# Save the plot as a TIFF file

tiff("Comparison_of_BAG_with_Significance.tiff", width = 6, height = 4, units = "in", res = 300)

print(plot)

dev.off()

# Create the scatter plot btn BAG_CH and FMA_2 with regression line

scatter_plot <- ggplot(data_1, aes(x = BAG_CH, y = FMA_2)) +

geom_point(color = "black", size = 3, alpha = 0.7) + # Points with color and size

geom_smooth(method = "lm", se = FALSE, color = "black", linetype = "solid", size = 1.2) + # Regression line

labs(

title = "",

x = "BAGCH (years)",

y = "Motor Outcome"

) +

theme_minimal() + # Clean minimal theme

theme(

plot.title = element_text(hjust = 0.5, size = 16, face = "bold", color = "black"),

axis.title = element_text(size = 20, face = "bold", color = "black"),

axis.line = element_line(color = "black", size = 1), # Black and thicker axis line

axis.text = element_text(size = 20, face = "bold", color = "black"),

panel.grid = element_blank() # Remove grid lines

)

# Display the plot

print(scatter_plot)

ggsave(

filename = "scatter_plot_OUTCOME.jpeg", # or .tiff, .jpeg, etc.

plot = last_plot(), # or specify your plot object, e.g., plot = p

width = 4, # width in inches (adjust as needed)

height = 4, # height in inches (adjust as needed)

units = "in",

dpi = 300

)
